# Aberrant Tryptophan Metabolism Manipulates Osteochondral Homeostasis

**DOI:** 10.34133/research.0728

**Published:** 2025-06-10

**Authors:** Tingwen Xiang, Chuan Yang, Langlang Xie, Shiyu Xiao, Yong Tang, Gang Huang, Dong Sun, Yueqi Chen, Fei Luo

**Affiliations:** ^1^ Department of Orthopedics, Southwest Hospital, Third Military Medical University (Army Medical University), Chongqing 400038, People’s Republic of China.; ^2^ Department of Biomedical Materials Science, Third Military Medical University (Army Medical University), Chongqing 400038, People’s Republic of China.; ^3^ Department of Biochemistry and Molecular Biology, College of Basic Medical Science, Third Military Medical University (Army Medical University), Chongqing 400038, People’s Republic of China.; ^4^ Department of Orthopedics, 76^th^ Group Army Hospital, Xining 810000, People’s Republic of China.

## Abstract

Tryptophan (Trp), an essential amino acid, performs as a precursor for synthesizing various bioactive molecules primarily metabolized through the kynurenine (Kyn), serotonin, and indole pathways. The diverse metabolites were deeply implicated in multiple physiological processes. Emerging research has revealed the multifaceted contribution of Trp in skeletal health and pathophysiology of bone-related disease with the involvement of specific receptors including aryl hydrocarbon receptor (AhR), which modulated the downstream signaling pathways to manage the expression of pivotal genes and thereby altered cellular biological processes, such as proliferation and differentiation. Accompanied by distinct alterations in immune function, inflammatory responses, endocrine balance, and other physiological aspects, their impact and efficacy in osteochondrogenic disorders have also been well documented. Nevertheless, a thorough understanding of Trp metabolism within bone biology is currently lacking. In this review, we elucidate the complexities of Trp metabolic pathway and several metabolites, delineating their versatile modulatory roles in the physiology and pathology of osteoblasts (OBs), osteoclasts (OCs), chondrocytes, and intercellular coupling effects, as well as in the progression of osteochondral disorder. Moreover, we comprehensively delineate the regulatory mechanisms by which gut microbiota-generated indole derivatives mediate bidirectional crosstalk along the gut–bone axis. The establishment of an elaborate governing network about bone homeostasis provides a novel insight on therapeutic interventions.

## Introduction

Tryptophan (Trp) is an essential amino acid that should be provided through diet, which is vital for whole-body homeostasis, particularly in regulating immune responses, maintaining redox equilibrium, and influencing nervous system function [1,2]. Apart from participating in the biosynthesis of macromolecular substances, Trp is metabolized through multiple pathways, leading to the production of various bioactive metabolites that dramatically exert biological effects throughout the body through circulation. The degradation of Trp primarily follows 3 metabolic pathways: (a) The kynurenine (Kyn) pathway involves the metabolism of Trp into Kyn catalyzed by the rate-limiting enzyme indoleamine 2,3-dioxygenase (IDO) and Trp-2,3-dioxygenase (TDO) [3]; (b) the serotonin (5-hydroxytryptamine) (5-HT) pathway entails Trp converting into serotonin in the gut and brain [peripheral 5-HT is mainly produced by Trp hydroxylase 1 (TpH_1_) in intestinal chromaffin cells]; (c) gut microbiota metabolizes Trp to produce indole-containing compounds and poly-aromatic hydrocarbon compounds in the intestinal cavity [4,5].

As a biosynthetic precursor of critical metabolites, Trp modulates various pathophysiological processes through complex downstream regulatory mechanisms. The Kyn pathway is of central importance in alleviating hyperinflammation and establishing long-term immune tolerance, characterizing cytoprotective and immunomodulatory properties in multiple conditions such as neurologic, psychiatric, and inflammatory bowel diseases [6]. Endogenous enzyme-catalyzed Kyn and gut microbiota-generated metabolites, such as indole, indole derivatives, tryptamine, and skatole, are able to modulate intestinal microenvironment homeostasis and extra-intestinal tissue physiology by directly targeting the host transcription factor aryl hydrocarbon receptor (AhR) [7]. The activation of AhR impacts immune phenotypes, suggesting antimicrobial and anti-inflammatory roles by inducing interleukin-22 (IL-22) transcription [8] as well as mediating regulatory T cell (Treg) differentiation [9]. Furthermore, 5-HT contributes to regulating mood, sleep, appetite, and intestinal homeostasis. It could be regarded as a classical gut–brain axis signaling, the imbalance of which contributes to the pathogenesis of multiple psychiatric and neurodegenerative conditions [10].

Growing evidence supports that Trp and its metabolites may exert different physiological meanings synergistically maintaining bone homeostasis [11]. Recent research has reported the disturbances of Trp metabolites in osteoarthritis (OA) [12], along with the substantial association with identified microbiota biomarkers and osteoporosis (OP) [13,14]. The anabolic products of the Kyn pathway, such as picolinic acid (PICA), have represented an aggressive effect on skeletal bone and muscle. On the contrary, high levels of 3-hydroxykynurenine (3-HK) and anthranilic acid (AA) have been reported to negatively influence skeleton, reducing bone mineral density (BMD), as well as raising fracture risk [15]. Additionally, the gut microbiota–osteochondral axis has been established to perform mediated by a diverse array of microbial metabolites. It was well known that gut-derived metabolites, including short-chain fatty acids, secondary bile acids, and Trp catabolites, exert systemic effects on bone homeostasis through receptor-mediated signaling, epigenetic modulation, immune modulation, and inflammation attenuation [5]. Short-chain fatty acids such as butyrate have been reported to modulate bone remodeling by suppressing osteoclast (OC) activity and promoting osteoblast (OB) differentiation, while bile acids can also regulate bone metabolism through FXR and TGR5 signaling. Trimethylamine N-oxide may exacerbate bone loss by promoting inflammatory responses. These metabolites collectively underscore the multifaceted microbial influence on osteochondral health. The disruption of gut microbiota alters the tendency of Trp metabolism and damages the proportion of metabolic components, resulting in the occurrence and development of age-related bone loss. Gut microbiota-derived AhR ligand has excessive potential in regulating osteochondral destruction diseases by supervising immune system and reducing inflammation levels [16,17].

Herein, this review delves into the impact of Trp metabolites on osteochondral destruction diseases by examining the current metabolic pathways and downstream signaling pathway effects, especially the dual roles in regulating bone remodeling balance of osteoblastogenesis and osteoclastogenesis. We also provide a comprehensive understanding on gut microbiota-derived indole metabolites in mediating the gut–bone axis. Importantly, we critically evaluate therapeutic opportunities targeting these pathways, including IDO1 inhibitors, AhR modulators, and microbiota-directed interventions, while highlighting existing challenges in translational applications.

## Biochemical Properties and Metabolic Pathways of Trp

Trp is the only amino acid containing an indole structure, first isolated in the early 20th century, which is classified as a group of exogenous amino acids. Trp has multiple isomers, such as L-Trp and D-Trp, among which L-Trp widely exists in humans and animals. Trp plays diverse physiological functions in the human body, not only as a component of various proteins and peptides but also involving the production of several bioactive compounds through multiple biosynthetic pathways, mainly including the Kyn, 5-HT, and indole pathways [18].

### The Kyn pathway

Kyn metabolism represents the dominant catabolic pathway for Trp, leading to the metabolites including Kyn and various downstream degradation products. Trp undergoes conversion to N-formylkynurenine (NFK) via 3 rate-limiting enzymes—IDO1, IDO2, and TDO, following by deacylated to Kyn via arylformamidase. Among these, IDO is extensively expressed in several organs such as the brain, liver, and gastrointestinal tract, while TDO is predominantly found in the liver. In the intestine, the Kyn pathway is primarily mediated by the rate-limiting enzyme IDO1. Kynurenine aminotransferases (KATs) facilitate the transformation of Kyn to kynurenic acid (KYNA). Additionally, Kyn can be converted to ortho AA by kynureninase (KYNU) or alternatively to 3-HK under the action of kynurenine 3-monooxygenase (KMO). KAT catalyzes 3-HK to xanthurenic acid (XANA), whereas KYNU mediates the conversion of 3-HK to alanine and 3-hydroxyanthranilic acid (3-HAA). Then, the former becomes quinolinic acid (QA), and the latter is converted to pyruvate by transamination. Kyn and its downstream metabolites are biologically active with multiple physiological functions. For instance, Kyn exerts inhibitory effects on the immune system [19], and meanwhile, nicotinamide adenine dinucleotide (NAD^+^) is involved in energy metabolism, calcium homeostasis, and gene expression [20].

### The 5-HT pathway

A small proportion of Trp is catabolized through the 5-HT pathway in the intestine and brain. TpH catalyzes Trp into 5-hydroxytryptophan (5-HTP), which is subsequently decarboxylated to form 5-HT under the aromatic acid decarboxylase (AADC). More than 90% of serotonin in the human body is produced in the intestine. Peripheral serotonin is predominantly biosynthesized by TpH_1_ in intestinal enterochromaffin cells, whereas the neurotransmitter 5-HT is produced in the brain via TpH_2_. Notably, under normal physiological circumstances, serotonin synthesized in the intestine is incompetent to pass through the blood–brain barrier or impact central nervous system function. In addition, 5-HT performs as substrate metabolized to produce N-acetylserotonin (NAS) by arylalkylamine N-acetyltransferase (AANAT) and then to melatonin by N-acetylserotonin O-methyltransferase (ASMT). Alternatively, with the enzyme monoamine oxidase (MAO), 5-HT can also be catalyzed into 5-hydroxyindoleacetic acid (5-HIAA). As a neurotransmitter, 5-HT may lead to neuropsychiatric disorders such as depression, anxiety, and social phobia during abnormal content and function in the central nervous system [21]. The multifaceted biological effects of 5-HT are mediated through its interaction with plasma membrane receptors, which are categorized into 7 distinct families comprising 16 specific subtypes. 5-HT_3_ (5-HT_3A_, 5-HT_3B_, and 5-HT_3C_) receptors belong to ionotropic receptor family, while others are recognized as parts of the G protein-coupled families, among which 5-HT_1_ (5-HT_1A_, 5-HT_1B_, 5-HT_1D_, 5-HT_1E_, and 5-HT_1F_) receptors suppress adenylate cyclase, 5-HT_2_ (5-HT_2A_, 5-HT_2B_, and 5-HT_2C_) receptors promote phospholipase C (PLC), and 5-HT_4_, 5-HT_6_, and 5-HT_7_ (5-HT_7A_, 5-HT_7B_, and 5-HT_7D_) receptors activate adenylate cyclase [22].

### The indole pathway

**Fig. 1.**
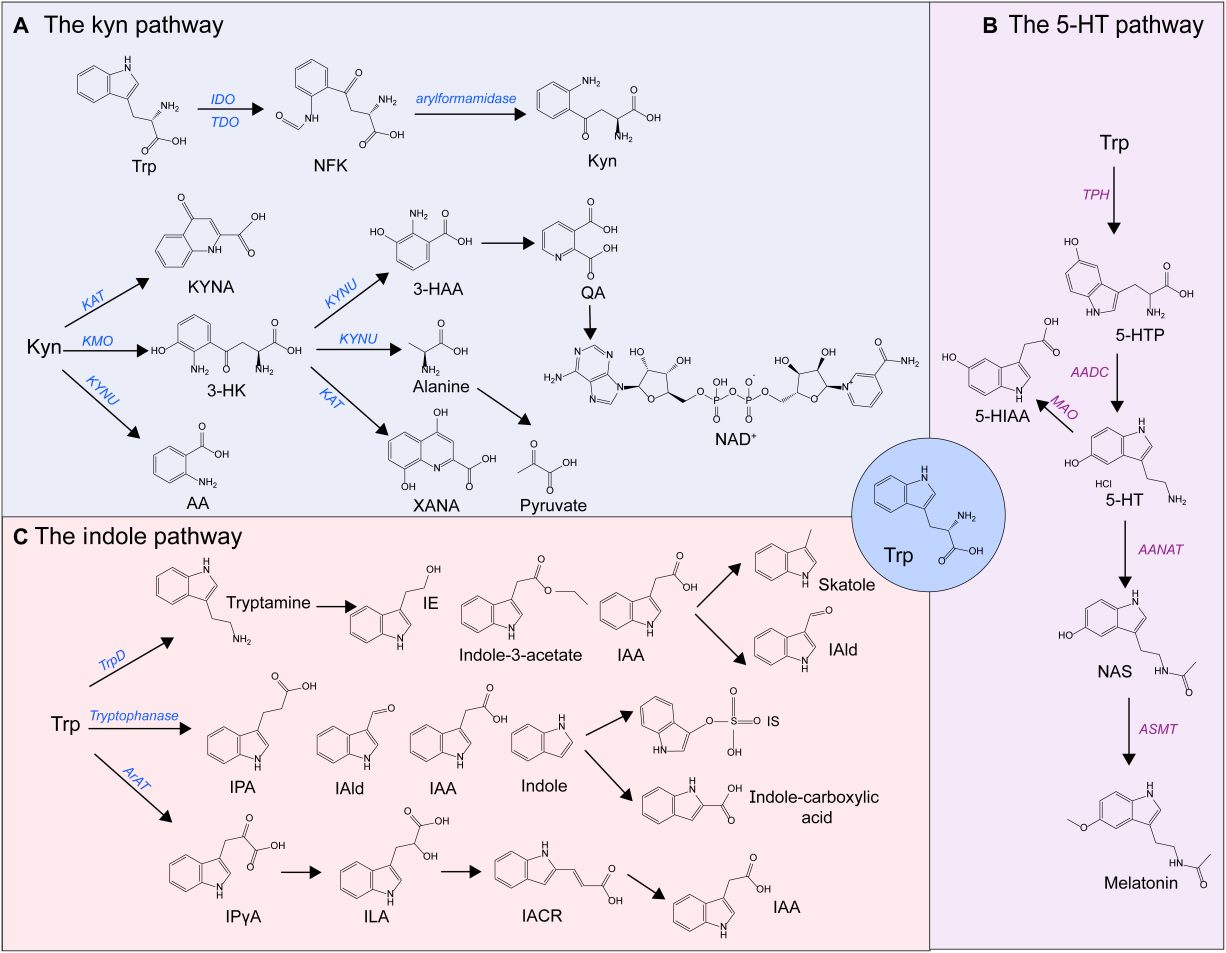
Three pathways of Trp metabolism in the human body. (A) Kyn pathway: Trp undergoes conversion to NFK via 3 rate-limiting enzymes—IDO1, IDO2, and TDO—and then is deacylated to Kyn by arylformamidase. Kyn can be converted to KYNA by KAT, AA by KYNU, or 3-HK by KMO. KAT catalyzes the conversion of 3-HK to XANA, whereas KYNU catalyzes it to 3-HAA and alanine, and then the former becomes QA, and the latter is converted to pyruvate through transamination. (B) 5-HT pathway: TpH catalyzes Trp into 5-HTP, which is decarboxylated to form 5-HT under AADC. 5-HT produces NAS by AANAT, then to melatonin by ASMT. Alternatively, 5-HT can also be catalyzed into 5-HIAA by MAO. (C) Indole pathway: TrpD catalyzes Trp to tryptamine, further processed into IE, indole-3-acetate, and IAA. Skatole and IAld are identified as the metabolites of IAA. Tryptophanase converts Trp into IAld, IAA, IPA, and indole. Indole can be further metabolized into IS and indole-3-carboxylic acid. Trp converts into IPγA mediated by ArAT, which performs as a precursor of ILA, IAcr, and IAA. [All original elements used in the schematic figures are acquired from Servier Medical Art (http://smart.servier.com/).]

Another pathway of Trp metabolism is the involvement of intestinal microbiota, contributing to the production of tryptamine, indole-3-propionic acid (IPA), indole-3-acetic acid (IAA), indole-3-aldehyde (IAld), indole-3-lactic acid (ILA), indole, and indole-3-pyruvate (IPγA). It was established that the gut microbiota performs as a part of endocrine system, producing various metabolites that play a crucial role in influencing host metabolism, homeostatic processes, and inflammatory responses, among which Trp serves as a substantial cross-kingdom metabolic substrate [23,24]. The enzyme Trp dehydrogenase (TrpD) catalyzes the conversion of Trp into tryptamine, which would be further processed into indole-3-ethanol (IE), indole-3-acetate, and IAA. Beyond that, Skatole and IAld are identified as the metabolites of IAA. Tryptophanase produced by microbiota converts Trp into IAld, IAA, IPA, and indole. Indole, which is absorbed through intestinal epithelium and enters the bloodstream, can be further metabolized into indoxyl in the liver and then processed into indole-3-carboxylic acid and indoxyl sulfate (IS). The aromatic amino acid aminotransferase (ArAT) contributes to converting Trp into IPγA, which performs as a precursor of ILA, indoleacrylic acid (IAcr), and IAA [25] (Fig. 1). Gut microbiota-derived indole and its derivatives are described as signaling molecules communicating with host cells. The influence of indole production on glucagon-like peptide 1 (GLP-1) exhibits time and spatial dependencies in enteroendocrine L cells, showing a positive effect during short exposures but became suppressive over longer periods [26]. Several metabolites, such as IPA and IAA, are known to improve intestinal barrier function, reverse the intestinal permeability, and maintain glucose homeostasis [27–29].

Specifically, extensive experimental research has clarified the targeted receptors of bioactive molecules and the downstream mechanisms. Several Trp catabolites perform as ligands for AhR in intestinal and extraintestinal cells. As a ligand-regulated transcription factor, AhR is translocated into the nucleus after being activated by an agonist, forming heterodimers with the AhR nuclear translocator (ARNT) protein, which subsequently interacts with the dioxin/xenobiotic response element (DRE/XRE) in the promoter sequences of AhR-responsive genes [30], leading to modulation of immune and inflammatory responses in a specific manner, as well as playing a crucial role in gut–brain bidirectional interactions [31–34]. Indole derivatives develop their biological effects through both the pregnane X receptor (PXR) and AhR [32,35]. Additionally, Kyn is also an agonist for AhR, and correspondingly, AhR provides a feedback regulation, reflected in the expression and activation of IDO, TDO2, KYNU, and KMO, which participate in regulating the Kyn metabolism [33].

Trp catabolites can exert influence on physiological and pathogenic status in various aspects. Obese individuals exhibit a higher Kyn/Trp ratio and lower levels of 5-HT and indoles, which are indicative of the associations with systemic inflammation [36]. The elevated production of Kyn results in reduced serotonin synthesis and may contribute to the susceptibility to certain psychiatric disorders, such as depression [37]. Given its pleiotropic functions in multiple pathological and physiological activities, how Trp metabolism is associated with bone homeostasis is a project worthy of in-depth research.

## Regulation of Bone Matrix Microenvironment by Trp Metabolites

Bone is a highly active biological structure composed of bone cells, bone matrix, and bone marrow. Bone homeostasis is a dynamic equilibrium state where bone tissue maintains its stability through continuous remodeling, which involves precise interactions between OBs, OCs, bone marrow cells, and other bone-related cells, as well as various biochemical signals within the bone microenvironment. The maintenance of bone homeostasis mainly depends on the equilibrium between OB-regulated bone formation and OC-driven bone resorption. OBs, derived from mesenchymal stem cells (MSCs), govern the deposition and mineralization of bone matrix, during which proteins related to OB phenotype such as osteocalcin (OCN), alkaline phosphatase (ALP), and osteopontin (OPN) are generated and released [38]. OCs are large multinucleated cells arising from monocytic progenitors in the bone marrow, initiated by macrophage colony-stimulating factor (M-CSF) and receptor activator of nuclear factor κB ligand (RANKL), and have a unique biochemical property to absorb mineralized matrix by synthesizing proteases including cathepsin K, tartrate-resistant acid phosphatase (TRAP), matrix metalloproteinases (MMPs), and hydrochloric acid [39,40]. The bone microenvironment, comprising bone matrix, growth factors, cytokines, and hormones, supports bone cell growth, differentiation, and activity. Recently, increasing evidence has proven that the role of Trp and its metabolites in bone homeostasis not only is limited to substrates for producing protein but also directly or indirectly influences the differentiation and activity of bone-related cells at multiple levels, such as gene level, transcriptional level, posttranscriptional level, translational level, and posttranslational level.

### Osteoblasts

Trp and its catabolites performed as essential factors in the maintenance of bone dynamic balance by regulating OB differentiation and activity. Studies have shown that a Trp-free diet can lead to reduced body weight, BMD, as well as delays in femoral bone growth in rats [11]. Trp, screened from 22 amino acids in previous experiments, has been demonstrated to maintain the stemness of bone marrow-derived mesenchymal stromal cells (BMMSCs) both in vivo and in vitro, which dramatically improved the proportion of SSEA-4-positive cells, mRNA levels of Nanog and Oct-4, and the migration and colony-forming ability of mouse BMMSCs. Additionally, L-Trp has been proven to promote osteogenic markers OPN and OCN in mRNA levels, as well as decreased mRNA levels of adipogenic markers peroxisome proliferator-activated receptor γ (PPAR-γ) and lipoprotein lipase (LPL), resulting in obvious enhancement of osteogenesis and bone regeneration [41].

In addition to Trp itself, various small-molecule metabolites of Trp exerted regulatory effects on OBs through various pathways and mechanisms. L-Kyn, a major metabolite of L-Trp, has been reported to present similar consequences of L-Trp in inducing stemness and osteogenic differentiation of BMMSCs by increasing mRNA levels of OPN and OCN, accelerating new bone formation. IDO1 activity was required to ensure the activation of the Kyn pathway, which played a pivotal part in the commitment of human MSC (hMSC) into the OB lineage [41]. Contrarily, in the crowded queue, human BMD was inversely correlated with the ratio of serum Kyn to Trp [42]. Prolonged treatment of Kyn would exacerbate bone aging phenotypes characterized by reduced bone density and increased marrow adiposity [43]. Under 50 μM Kyn, the expressions of CYP1A1 and CYP1B1 were significantly up-regulated, indicative of oxidative stress, further supporting the ability to produce reactive oxygen species (ROS) in bone marrow stromal cells [44]. Moreover, in vitro experiments showed that Kyn treatment hindered the capacity of bone marrow stromal cell–OB cultures to generate calcified matrix and repressed the expression of Runt-related transcription factor 2 (Runx2), which may be partially due to impaired osteoblastic bioenergetics, specifically the damaged production of adenosine triphosphate (ATP) through oxidative phosphorylation during cellular stress [42]. Additionally, Kyn could up-regulate miR-29b-1-5p to decrease the expression of CXCL12 and the cognate receptors ACKR3 and CXCR4 partially through the AhR pathway, which resulted in a notable inhibition of osteogenic differentiation in bone marrow stromal cells [45]. The effect of Kyn on osteogenesis is still controversial, so it will be necessary to determine the actual clinical effect and the mechanisms by which Kyn contributes to bone metabolism from multiple perspectives. Furthermore, regarding other metabolites, PICA has also been demonstrated to develop a proactive impact on bone formation in vivo. Mechanistically, it significantly promoted osteogenic gene expression such as Runx2 and OCN with a dose-dependent osteogenic effect [46,47].

5-HT and its derivatives performed important biological functions as regulators of bone metabolism. The present studies have reported that 5-HT obstructed OB functions including proliferation, differentiation, and mineralization at low concentrations; however, at elevated concentrations, the disincentive was attenuated, even reversed. The bidirectional regulation of 5-HT on bone homeostasis may be attributed to the distinct downstream signaling pathways of specific receptor subtypes [48]. Among the universal 5-hydroxytryptamine receptor (5-HTR), the three (HTR_1B_, HTR_2B_, and HTR_2A_) were remarkably expressed in OBs. Enterochromaffin cell-derived 5-HT can act as a hormone by targeting the HTR_1B_, which belongs to the Gαi protein-coupled receptor (GPCR). The activation of HTR_1B_ diminished phosphorylation of cAMP response element binding (CREB) on Ser^133^ and restrained binding of CREB to the promoter of CyclinD1, thereby inhibiting OB proliferation [49]. Nevertheless, HTR_2a_ and HTR_2b_, which are part of the Gαq/11-GPCR family, transmit signals through the PLC–inositol phosphate 3/diacylglycerol–protein kinase C (PLC-IP3/DAG-PKC) signaling pathway to enhance OB proliferation and bone formation [50]. It was hypothesized that a low concentration of 5-HT enabled HTR_1B_, resulting in the inhibition of proliferation of OBs, and conversely, higher concentrations may primarily activate HTR_2A_ and HTR_2B_ to exploit the opposite effect. In comparison to gut-derived serotonin, brain-derived serotonin produced by serotonin neurons of the hindbrain functioned as a neurotransmitter and played an aggressive role in the central nervous system [51]. Additionally, melatonin, a metabolite of 5-HT, exerted beneficial actions in preventing oxidative stress-inhibited osteogenesis. Melatonin has been shown to counteract the inhibitory effects of tumor necrosis factor-α (TNF-α) on osteogenic differentiation and inflammation [52]. Melatonin significantly facilitated the osteogenic differentiation of bone marrow mesenchymal stem cells (BMSCs) by curbing the melatonin receptor 1B (MT_2_)-mediated nuclear factor κB (NF-κB) signaling pathway [53]. Melatonin administration also promoted osteogenic differentiation of hMSCs by activating adenosine monophosphate-activated protein kinase (AMPK) and up-regulating the master transcription factors such as Foxo3a and Runx2 that established the mechanistic connection between oxidative stress and osteoblastic behavior [54]. Besides, melatonin possessed the ability to facilitate Osterix expression through protein kinase A (PKA) and PKC signaling pathways, as well as restrain the degradation mediated by ubiquitin proteasome, resulting in enhanced Osterix transcriptional activity on the osteogenic promoter, which in turn promoted bone mineralization [55]. Furthermore, the study also substantiated that melatonin selectively promoted ZIP-1 to increase citrate and mineralize nodules in OBs derived from primary mouse BMSCs in vitro, which may alleviate bone mass by boosting matrix mineralization [56]. In the ovariectomy (OVX) animal model, pineal-derived melatonin has been verified to modulate OB proliferation, activity, and function, as well as restore bone loss through MT_2_ [57]. Regarding epigenetics, melatonin could suppress the expression of circ_0003865 to regulate GAS1 translationally by sponging miR-3653-3p, therefore leading to the enhancement of BMSC osteogenic differentiation [58]. Melatonin also promoted the expression of the histone methyltransferase nuclear receptor binding SET domain protein 2 (NSD2) by targeting to MT_1/2_, which contributed to the management of H3K36me2 and H3K27me3 modification, thereby increasing osteogenic gene chromatin dynamics including Runx2 and bone γ-carboxyglutamate protein (BGLAP) [59].

Multiple products of the indole metabolism pathway participated in regulating bone anabolism via the gut–bone axis. Gut microbial-derived IPA has also been elucidated to improve OB mineralization in obese mouse models. Mechanistically, probiotics or IPA treatment reduces repressive H3K27me3 epigenetic methylation at the mitochondrial transcription factor A promoter by promoting Kdm6b/Jmjd3 histone demethylase, thereby boosting mitochondrial function and osteogenesis [60]. Furthermore, a 2025 review comprehensively summarized that IPA not only directly regulated bone homeostasis by modulating the expression of OB- and OC-related genes but also exerted indirect skeletal protection through receptor-mediated modulation of host immune responses and inflammatory processes [61]. IS, metabolized from Trp, was identified as a uremic toxin, which inhibited extracellular signal-regulated kinase (ERK) and p38 mitogen-activated protein kinase (MAPK) pathway through the AhR signaling. The suppression of upstream signaling modulated the expression of Runx2, ultimately interrupting osteoblastogenesis [62]. The AhR antagonist resveratrol (RSV) has been proven to exert a preservative effect on the IS/AhR/MAPK pathway to rescue IS-induced osteoblastogenesis exacerbation in chronic kidney disease patients [63].

### Osteoclasts

Trp metabolites regulate OC functionality and the process of bone resorption through a sophisticated signaling network. The oxidized form of L-Trp encouraged bone marrow-derived stem cells to differentiate into OBs, while in contrast, Kyn regulated OC activity and facilitated the evolution of bone marrow-derived stem cells into adipocytes [64]. Moreover, gradually accumulating kyn contributed to osteoblastic differentiation disruption as well as boosting OC-governed bone resorption, thereby expediting skeletal aging [43], where the AhR genomic pathway has been involved in RANKL-induced osteoclastogenesis through the up-regulation of c-Fos and nuclear factor of activated T cells, cytoplasmic 1 (NFATc1) [65]. Nevertheless, there remains controversy over this issue. AhR was regarded as a dual effector on bone homeostasis. The diversity of downstream signaling pathways contributed to the complexity of its effects. Depending on its role as either a ligand-activated transcription factor or an E3 ubiquitin ligase, AhR can either facilitate or impede OC differentiation. AhR enhanced OC differentiation and activity by activating RANK/c-Fos signaling and NF-κB pathways, and promoting Blimp1, Cyp1b1, and Cyp1a2 expression [66–68]. Conversely, more recently, the inhibitory function of AhR activation toward OC differentiation in human cells has been certified. Through a nongenomic mechanism, AhR was involved in obstructing OC differentiation by inducing the proteasomal degradation of NFATc1 and Syk [69]. Specifically, Kyn inhibited human OC differentiation via post-transcriptional regulation of NFATc1, specifically targeting protein expression rather than mRNA, which indicated that AhR possessed the ability to perform as a potential therapeutic molecule within bone destruction diseases in clinical practice [70]. Under the condition of AhR knockout, the OCs derived from bone marrow were significantly inhibited, while in another article, the opposite conclusion was drawn after conducting receptor blockade experiments treated by AhR inhibitor CH223191 at 5 μM in peripheral blood mononuclear cells (PBMCs). The discrepancy in AhR regulation across different studies may be attributed to several factors, including the specific ligand employed to activate AhR signaling, the endurance of AhR ligand binding, the composition of transcriptional complexes, and the biological models utilized. Overall, the Kyn-AhR system represents a new research pathway that requires additional exploration to tackle bone deterioration in diseases related to aging.

5-HT has been recently determined as a critical modulator of bone turnover. In human PBMCs that have differentiated into OCs, the existence of 5-HTR_2A,B,C_ is evident [71]. TpH_1_, 5-HTT, and 5-HTR_1B_ were also expressed in OCs, while the expression of 5-HTR_2B_ notably increased as precursor cells mature into OCs [72]. Intracellular 5-HTR_6_ signaling was linked to RhoA guanosine triphosphatase (GTPase) activation and OC maturation [73]. Serotonin may function through both autocrine and paracrine signaling between OBs and OCs. RANKL promoted TpH_1_ expression and the level of OC-synthesized serotonin. The serotonin generated by osteoclast precursors (OCPs) could collaborate in concert with RANKL signaling to further advance OC differentiation [74]. Under the condition of periodontitis, osteoclastogenesis was also stimulated by 5-HTP, which raised the RANKL/osteoprotegerin (OPG) ratio and the quantity of IL-6^+^ osteocytes, thereby exacerbating the loss of alveolar bone and worsening the microstructure [75]. Additionally, metabolites may contribute to stabilizing BMD. In experimental models of pulmonary and prostatic osseous metastases, melatonin has been discovered to significantly alleviate osteolytic process. Mechanistically, melatonin directly diminished the percentage of TRAP^+^ OCs in the tibia bone marrow and also dramatically suppressed RANKL manufacturing in lung and prostate cancer cells through the inactivation of the p38 MAPK pathway, subsequently restraining cancer-associated OC differentiation [76]. Within the coculture of BMSCs and OCP, melatonin attenuated BMSC-mediated osteoclastogenesis through down-regulation of RANKL expression in an indirect contact manner [53]. Melatonin manifested the disincentive on OCs by accelerating osteocyte-secreted calcitonin in chick calvariae [77]. In RAW264.7 cells, melatonin also played an important role in inhibiting osteoclastogenesis through the miR-882/Rev-erbα axis under the treatment of RANKL and M-CSF [78]. Apart from involving noncoding RNA (ncRNA), the mechanism of repression may refer to the inhibition of the NF-κB signaling pathway. Furthermore, at pharmacological doses, melatonin effectively suppressed the osteoclastogenesis of bone marrow monocytes (BMMs) via a ROS-mediated pathway [79]. Fluoxetine (Flx), recognized as one of the most typically prescribed selective serotonin reuptake inhibitors (SSRIs), possessed anti-resorptive characteristics. Both serotonin and Flx can influence the formation of OBs and OCs in vitro, with effects that can be either beneficial or detrimental depending on their concentrations. At micromolar concentrations, flx restrained OC differentiation and activity, while at nanomolar levels, there appeared to be an enhancement in OC activation [71]. Moreover, Flx exhibited a dual effect in time-dependent mechanisms in murine models, which directly hindered the differentiation and functionality of OCs via a serotonin reuptake-independent manner, but rather relied on intracellular Ca^2+^ levels and the transcription factor NFATc1. Over time, Flx additionally stimulated a serotonin-dependent increase in sympathetic output from the brain, which significantly enhanced bone resorption [80].

Gut *Clostridium sporogenes-*derived IPA was implicated in the estrogen deficiency-induced OC overactivation, which suppressed the ubiquitination and decomposition of PXR to enhance PXR/P65 complex synthesis, thereby mitigating bone loss elicited by OVX [81]. Moreover, during the process of osteoclastogenesis, IS affected the NFATc1 expression in OCP mediated by AhR signaling pathways in a time-dependent manner. Exposure to IS at short durations and low doses exerted a positive function in OC differentiation, whereas prolonged exposure or high doses of IS might lead to a reduction [62].

### Chondrocytes

Chondrocyte is the primary cell type that constitutes cartilage tissue, mainly responsible for synthesizing and secreting the principal substances of the cartilage matrix, such as collagen and proteoglycans, which confer the unique elasticity and strength characteristic of cartilage tissue. Recent studies have demonstrated that Trp metabolism participated in adjusting the structure and function of cartilage. Trp metabolites, particularly Kyn, impaired the chondrogenesis and chondroprotective utility of human umbilical cord mesenchymal stem cells (hUC-MSCs) by activating the AhR pathway [82]. Besides, septic arthritis was also pertinent to elevated levels of synovial Kyn due to its effects on suppressing the proliferation of ATDC5 cells in a dose-dependent manner [83].

5-HT contributed to the processes of cartilage development and regeneration. Studies have identified the expression of 5-HT_2A_R in the growth plate, while 5-HTR_2B_ was present in the articular cartilage. In the growth plates, 5-HT facilitated CCN2 production engaged by 5-HTR_2A_, whereas it reduced CCN2 generation via 5-HTR_2B_ in articular cartilage to facilitate coordinated growth of long bones [84]. Additionally, 5-HT can also stimulate phospholipase A2 in a dose-dependent manner to increase the activity of collagenase type II, causing aggravated damage to cartilage [85]. Research indicated that melatonin administration resulted in heightened expression of markers indicative of chondrocyte differentiation, supporting the conducive function of melatonin in the hMSC-derived chondrogenic differentiation [86]. Further research explored that melatonin appears to facilitate the synthesis of the cartilage matrix in articular chondrocytes via the transforming growth factor-β (TGF-β) signaling pathway within a pellet culture system containing serum [87]. Melatonin also rescued IL-1β-damaged chondrogenesis of human BMSCs in multiple manners including recovering pellet size and matrix accumulation, sustaining the metabolic balance by down-regulating the expression of catabolic genes, such as MMP-13 and ADAMTS4, and promoting chondrogenic marker collagen type II α1 (Col2A1) expression transcriptionally and translationally, as well as subduing cell apoptosis. Moreover, melatonin has been confirmed to repress the phosphorylation level of P65 and IκBα, dampening the activation of the NF-κB signaling pathway [88]. Additionally, melatonin played an antagonistic role in the MAPK signaling pathway, which repressed phosphorylation of ERK1/2 to inhibit the secretion of pro-inflammatory factors including IL-1β and TNF-α [89]. However, some results revealed completely contrasting data regarding the impact of melatonin on chondrocyte differentiation. Melatonin may promote chondrogenic differentiation and hypertrophy in MSC-derived chondrocytes via stimulation of the Wnt/β-catenin cascade, culminating in β-catenin nuclear accumulation. The discrepancy could originate from the cellular heterogeneity among different chondrocyte types [90]. Furthermore, melatonin has been demonstrated to safeguard chondrocytes through the maintenance of mitochondrial redox balance and the process of autophagy. Chen et al. also explored how melatonin exhibited anti-apoptotic properties and autophagy effects that shield rat chondrocytes from oxidative stress by modulating the AMPK/Foxo3 pathway [91]. Additionally, melatonin enhanced the expression of Silent information regulator type 1 (Sirt1) while simultaneously inhibiting IRE1α-XBP1S-CHOP (C/EBP homologous protein), thereby alleviating apoptosis induced by endoplasmic reticulum (ER) stress (ERS) in chondrocytes [92]. The upsurge in Sirt1 expression and activity also curtailed endplate chondrocyte (EPC) calcification in a concentration-dependent manner, promoting autophagic processes in EPCs [93]. The further examination of Dex-induced matrix degradation in chondrocytes also proved the chondroprotective properties of melatonin related to NAD^+^-dependent Sirt1 promotion [94]. Notably, high concentrations of melatonin impeded chondrocyte proliferation and differentiation in vertebral body growth plate (VBGP), down-regulated the collagen type II (Col2) and aggrecan expression, as well as reduced the protein expression levels of proliferating cell nuclear antigen (PCNA), Sox9, and Smad4 [95]. Beyond these, melatonin directly influenced the circadian rhythms of chondrocytes. Both exogenous and endogenous melatonin cooperated within chondrocytes to synchronize the rhythmic expression with the central clock located in the suprachiasmatic nucleus. Chondrocytes were capable of producing melatonin, regulating the growth and maturation of cartilage through MT_1_ and MT_2_. The presence of melatonin led to a swift elevation of Aanat, Mt1, Mt2, and Pthrp expression, subsequently followed by increased levels of Sox9 and Ihh. Besides, the expression of the clock gene Bmal1 was enhanced, whereas Per1 expression was reduced. Melatonin also triggered the rhythmic expression of Aanat and altered the cyclic rhythm of Bmal1, Mt1, and Mt2 [96]. Epigenetic mechanisms also intricately governed the impact of melatonin on chondrocyte activity, shaping the development and functional dynamics in a nuanced manner. Melatonin up-regulated the expression of miR-526b-3p and miR-590-5p, which in turn boosted the phosphorylation of SMAD1 by targeting SMAD7, ultimately promoting the chondrogenic differentiation of human BMSCs [97].

**Fig. 2.**
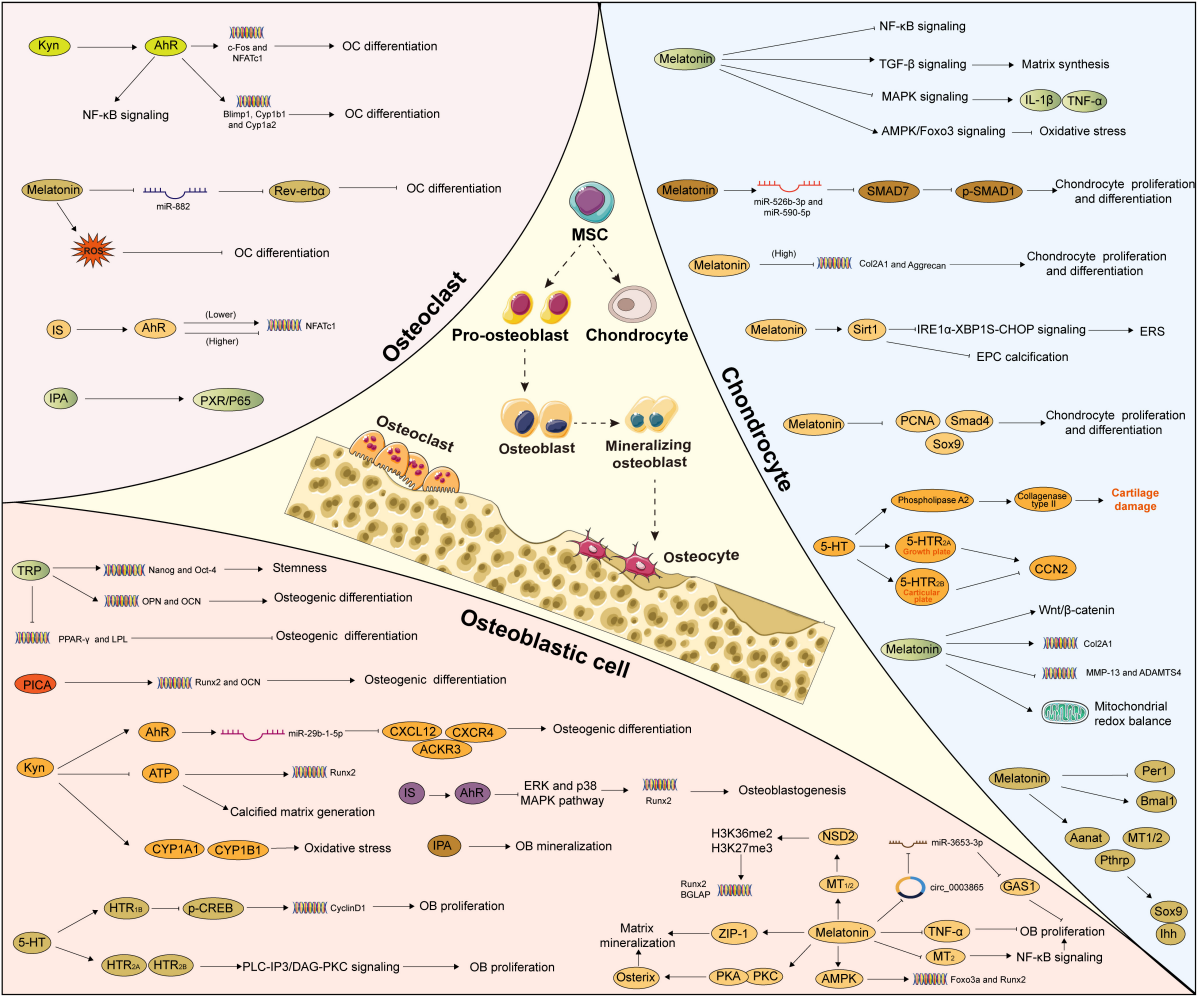
The bioactive effect of Trp metabolites in bone-related cells. Osteoblastic cell: Trp improved mRNA levels of Nanog and Oct-4 to maintain stemness, up-regulated osteogenic markers OPN and OCN, and decreased PPAR-γ and LPL to promote osteogenesis. Kyn up-regulated CYP1A1 and CYP1B1 expression to generate ROS. Kyn inhibited Runx2 expression and generated calcified matrix by damaging ATP production. Kyn could up-regulate miR-29b-1-5p to decrease CXCL12, CXCR4, and ACKR3 partially through the AhR pathway to inhibit osteogenic differentiation. PICA promoted the expression of osteogenic genes Runx2 and OCN to protect bone formation. 5-HT activated HTR_1B_ to diminish phosphorylation of CREB and suppress CyclinD1. 5-HT also targets HTR_2a_ and HTR_2b_ to activate the PLC-IP3/DAG-PKC signaling pathway to enhance OB proliferation. Melatonin conversed the inhibitory effects of TNF-α on osteogenic differentiation, curbed the MT_2_-mediated NF-κB signaling pathway, and activated AMPK signaling, up-regulating Foxo3a and Runx2. Melatonin facilitated Osterix expression through PKA and PKC signaling pathways and up-regulated ZIP-1 to promote bone mineralization. Melatonin could suppress the expression of circ_0003865 to regulate GAS1 translationally by sponging miR-3653-3p to enhance osteogenic differentiation. Melatonin also promoted NSD2 expression by targeting MT_1/2_ to manage H3K36me2 and H3K27me3 modification, increasing Runx2 and BGLAP expression. IS inhibited ERK and p38 MAPK pathway through the AhR signaling. Osteoclastic cell: Kyn activated AhR to up-regulate c-Fos and NFATc1, as well as Blimp1, Cyp1b1, and Cyp1a2 expression, and enhanced NF-κB pathways to promote OC differentiation. Melatonin inhibited osteoclastogenesis through the miR-882/Rev-erbα axis and induced ROS. IPA enhanced PXR/P65 complex synthesis. IS affected the NFATc1 expression mediated by AhR signaling pathways in a time- and dose-dependent manner. Chondrocyte: 5-HT facilitated CCN2 production engaged by 5-HTR_2A_ in the growth plates and reduced CCN2 generation through 5-HTR_2B_ in articular cartilage. 5-HT can stimulate phospholipase A2 to increase collagenase type II activity, causing cartilage damage. Melatonin up-regulated Aanat, Mt1, Mt2, and Pthrp expression, subsequently followed by increased levels of Sox9 and Ihh. Besides, Bmal1 expression was enhanced, whereas Per1 expression was reduced. Melatonin facilitated the cartilage matrix synthesis via the TGF-β signaling pathway. Melatonin down-regulated the expression of MMP-13 and ADAMTS4 and promoted Col2A1 expression. Melatonin also inhibited the MAPK signaling pathway to repress IL-1β and TNF-α and was involved in activating the Wnt/β-catenin signaling pathway and inactivating the NF-κB signaling pathway. Melatonin maintained mitochondrial redox balance and repressed oxidative stress by modulating the AMPK/Foxo3 pathways. Melatonin enhanced the expression of Sirt1, inhibiting IRE1α-XBP1S-CHOP to alleviate ERS and curtailing EPC calcification. High melatonin concentrations impeded the chondrocyte proliferation and differentiation by down-regulating the Col2 and aggrecan expression and reduced the protein expression levels of PCNA, Sox9, and Smad4. Melatonin also up-regulated the expression of miR-526b-3p and miR-590-5p, which in turn boosted the phosphorylation of SMAD1 by targeting SMAD7, ultimately promoting chondrogenic differentiation. [All original elements used in the schematic figures are acquired from Servier Medical Art (http://smart.servier.com/).]

The endogenous ligand IPA could suppress inflammation caused by IL-1β and cartilage extracellular matrix (ECM) degradation, enhance matrix synthesis, and inhibit the NF-κB signaling pathway by targeting AhR in chondrocytes [98]. In a similar manner, IAld, a Trp metabolite secreted by intestinal flora, can also diminish IL-1β-induced inflammation through the AhR–NF-κB signaling pathway in chondrocytes [99] (Fig. 2 and Table 1).

**Table 1.** Trp metabolism pathways and the roles of metabolites in bone cells

Pathways	Metabolites	Structure	Recipient cells	Operations	References
	L-Trp	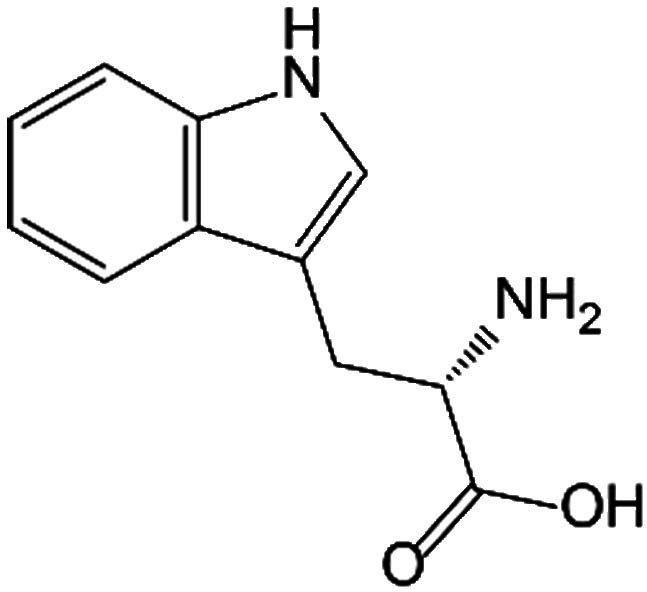	BMMSC	Promoted osteogenic markers OPN and OCN in mRNA levels, as well as decreased mRNA levels of adipogenic markers PPAR-γ and LPL, enhancing osteogenesis and bone regeneration.	[41]
Kyn pathway	Kyn	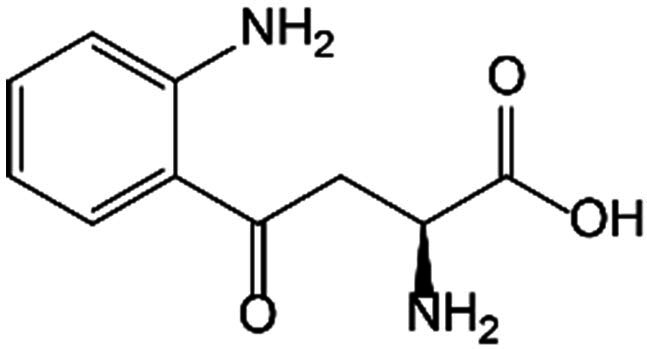	Bone marrow stromal cells	Suppressed the expression of Runx2.	[42]
				Up-regulated CYP1A1 and CYP1B1 expressions, supporting the generation of ROS.	[44]
				Up-regulated miR-29b-1-5p and decreased the expression of CXCL12, CXCR4, and ACKR3 to inhibit osteogenic differentiation.	[45]
			BMMSC	Promoted the expression of OPN and OCN to induce stemness and osteogenic differentiation.	[41]
			OB	Suppressed the expression of Runx2.	[42]
			OC	Controversial	[65,70]
5-HT pathway	5-HT	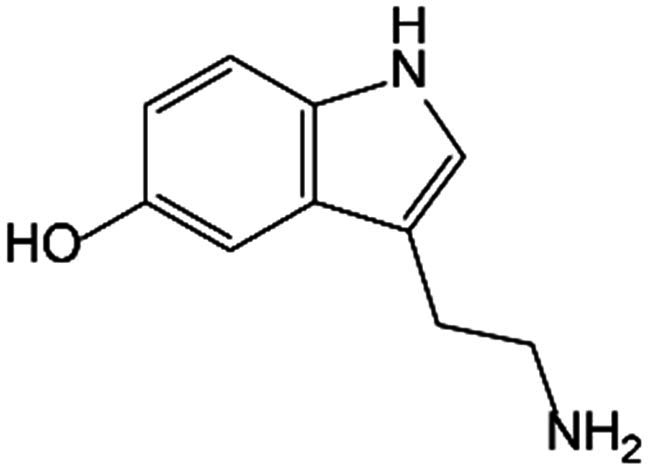	OB	Diminished CREB phosphorylation on Ser^133^ and restrains binding of CREB to CyclinD1 by activating HTR_1B_ to inhibit OB proliferation.	[49]
			Chondrocyte	Facilitated CCN2 production engaged by 5-HTR_2A_, but reduced CCN2 generation through 5-HTR_2B_.	[84]
				Stimulated phospholipase A2 in a dose-dependent manner to increase collagenase type II activity, causing aggravated cartilage damage.	[85]
	5-HTP	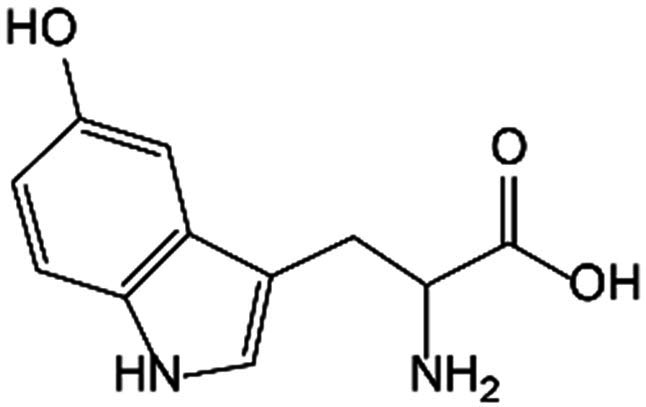	OC	Stimulated osteoclastogenesis.	[75]
	Melatonin	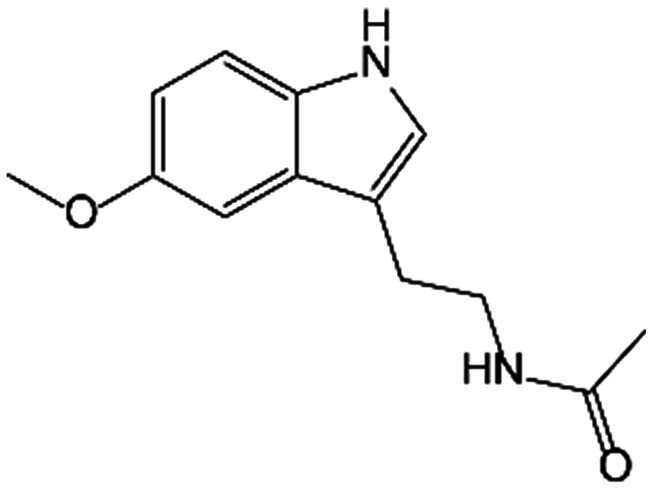	BMSC	Inhibited the osteogenic differentiation and inflammatory response by suppressing the NF-κB signaling pathways.	[52,53]
				Suppressed the expression of circ_0003865 to regulate GAS1 translationally by sponging miR-3653-3p to enhance osteogenic differentiation.	[58]
				Rescued IL-1β-damaged chondrogenesis of human BMSCs by down-regulating expression of catabolic genes, promoting chondrogenic marker, and subduing cell apoptosis.	[88]
				Repressed phosphorylation of ERK1/2 to inhibit the secretion of pro-inflammatory factors such as IL-1β and TNF-α.	[89]
				Up-regulated the expression of miR-526b-3p and miR-590-5p, which boosted the phosphorylation of SMAD1 by targeting SMAD7 to promote chondrogenic differentiation.	[97]
			Bone marrow stromal cells	Promoted the expression of the histone methyltransferase NSD2 to increase chromatin accessibility of osteogenic genes.	[59]
			MSC	Promoted osteogenic differentiation by activating AMPK and up-regulating the master transcription factors.	[54]
				Stimulated hypertrophy and the differentiation of MSC-derived chondrocytes by activating the Wnt/β-catenin signaling pathway and promoting the nuclear translocation of β-catenin.	[90]
			OB	Facilitated Osterix expression through PKA and PKC signaling pathways and enhanced the transcriptional activity to promote bone mineralization.	[55]
				Up-regulated ZIP-1 to increase citrate and mineralize nodules in OBs.	[56]
			Osteocyte	Accelerated osteocyte-secreted calcitonin to inhibit OC activity.	[77]
			Macrophage	Inhibited osteoclastogenesis through miR-882/Rev-erbα axis.	[78]
			Monocyte	Suppressed osteoclastogenesis via ROS-mediated pathway.	[79]
			OC	Restrained cancer-associated OC differentiation.	[76]
			Chondrocyte	Exhibited anti-apoptotic properties and autophagy effects that protected chondrocytes from oxidative stress through AMPK/Foxo3 pathways.	[91]
				Enhanced Sirt1 expression while inhibiting IRE1α-XBP1S-CHOP, thereby alleviating ERS-induced apoptosis.	[92]
				Reduced apoptosis and curtailed EPC calcification in a concentration-dependent manner, promoting Sirt1 expression and activity, and autophagic processes.	[93]
				High concentrations of melatonin impeded the proliferation and differentiation of chondrocytes in VBGP.	[95]
				Influenced the circadian rhythms.	[96]
Indole pathway	IS	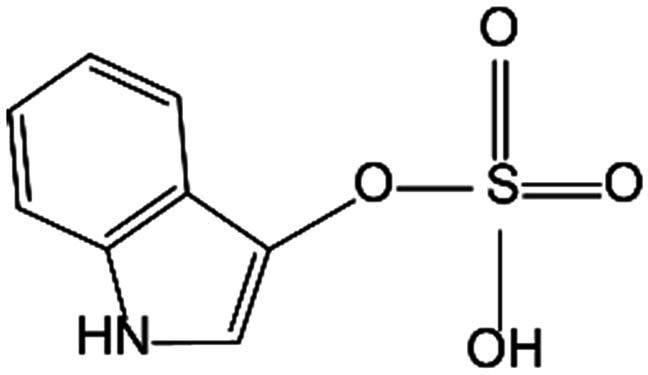	OB	Inhibited ERK and p38 MAPK pathway through the AhR signaling to disturb osteoblastogenesis.	[62]
			OCP	Regulated NFATc1 expression in OCP mediated by AhR signaling pathways in a time-dependent manner.	[62]
	IPA	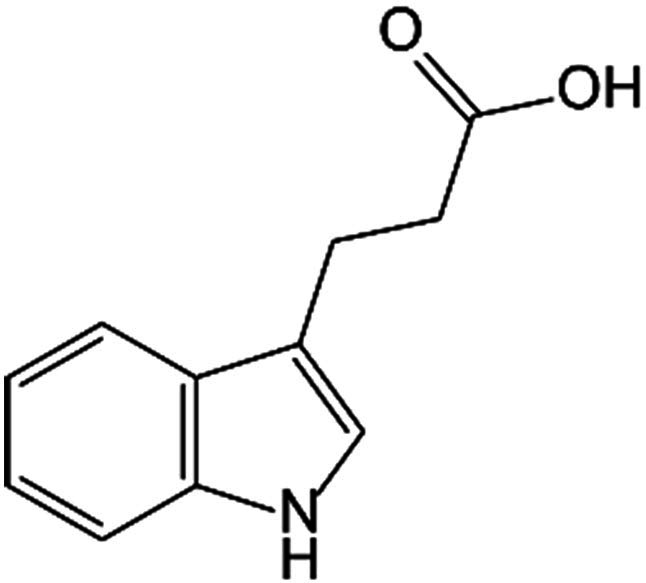	OB	Increased mitochondrial transcription factor A expression by promoting Kdm6b/Jmjd3 histone demethylase.	[60]
			OC	Suppressed the ubiquitination and decomposition of PXR to enhance PXR/P65 complex synthesis, thereby mitigating bone loss elicited by OVX.	[81]
			Chondrocyte	Suppressed IL-1β -induced inflammation and cartilage ECM degradation, enhanced matrix synthesis, and inhibited the NF-κB signaling pathway by targeting AhR.	[98]
	3-IAld	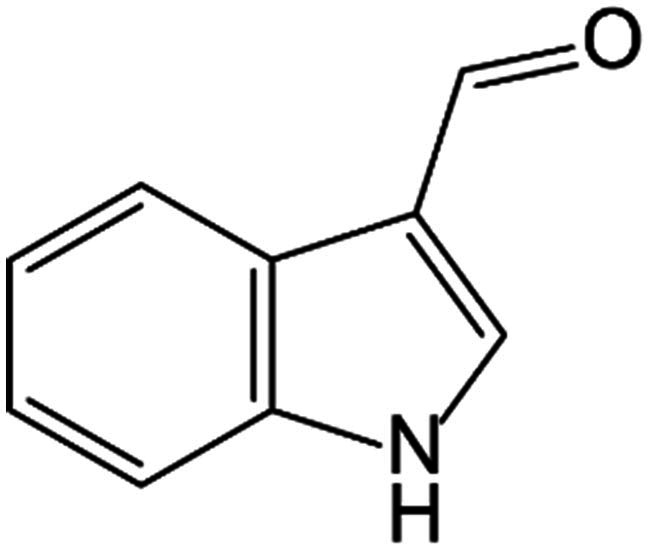	Chondrocyte	Diminished IL-1β-induced inflammation through the AhR-NF-κB signaling pathway.	[99]

### Multicellular coupling effects

The general function of bone tissue relies on intricate interactions among various cell types, a phenomenon commonly referred to as multicellular coupling effects. Within the bone microenvironment, cells such as OBs, OCs, and bone marrow mesenchymal cells coordinate through intercellular communication and signal transmission to collectively sustain bone health and stability [100,101]. Specifically, bone cells synchronize the regulatory function through direct cell-to-cell contact to rapidly alter the state of bidirectional transduction signals present on the bilateral cells such as Ephrin B2 (EFNB2)-EPHB4, FASL-FAS, or SEMA3A-NRP1 [102–104]. Simultaneously, released cytokines also govern crosstalk of bone cells. Soluble factors produced by OBs such as M-CSF, RANKL/ OPG, WNT5A, and WNT16, as well as sphingosine 1 phosphate, semaphorin 4D, collagen triple helix repeat containing 1, and complement component C secreted by OCs contribute to maintaining a dynamic balance between bone formation and resorption [105]. Furthermore, interactions with the ECM, including collagen and glycosaminoglycans, provide structural support for bone cells while also transmitting biomechanical signals that further regulate cellular function. Growth factors in the ECM, such as insulin-like growth factor 1 (IGF-1) and TGF-β1, promote cell proliferation, differentiation, and functional maintenance by binding to targeted receptors [106,107]. Collectively, the mechanisms demonstrated above ensure the effective self-repair and reshaping of bone tissue in response to physiological demands and environmental changes.

Research indicated that Trp metabolites were involved in regulating cellular behavior, survival, and differentiation by influencing multicellular coupling effects, intercellular signaling, and cellular functions. Kyn could promote the bone mineralization of OB differentiation as well as inhibit OC differentiation, during which the potential coupling factors linking OC and OB have also been demonstrated. Mechanistically, Kyn significantly increased the level of OPG proteins secreted by osteoprogenitors, which decreases TRAP-positive OC formation and NFATc1 expression, ultimately interrupting the RANKL-mediated osteoclastogenesis of OCP [108]. 5-HT may exert distinct effects on bone metabolism under healthy and diseased conditions. Specifically, serotonin has been shown to enhance the secretion of OPG from OBs while diminishing RANKL exudation, indicating its role in the OB-mediated inhibition of OC differentiation [71]. Conversely, other studies have reported that 5-HTP up-regulated the RANKL/OPG ratio produced by OBs in the presence of periodontitis, thereby contributing to osteoclastogenesis [75]. Besides, research has also demonstrated that osteocyte-secreted IL-6 could influence bone remodeling through soluble factors. 5-HT stimulated IL-6 secretion from osteocytes to promote osteoclastogenesis through the elevation of RANKL expression mediated by activating the gp130-STAT3 pathway in OBs and stromal cells [75].

Furthermore, various immune cells also interact with OBs and OCs through direct cell-to-cell contact or paracrine mechanisms [109]. Since the concept of “osteoimmunology” was proposed by Arron and Choi in 2000 [110], it has been substantiated that bone and immune cells shared the same microenvironment and interacted to collaboratively fulfill the functions of the “osteoimmune system”, encompassing all cells present in the bone marrow [111]. On the one hand, Trp metabolism may modulate the cytokines secreted by OBs, which in turn affect bone homeostasis through the modulation of immune cell functions and local immune environment. Previous research has reported that the increase in KYNA manufacturing could ameliorate inflammatory responses [112]. Meanwhile, KYNA has also been proven to restrain neutrophil activity and down-regulate inflammatory factors in a mouse model of sepsis [113], which may result in preventing exacerbation of bone resorption [114]. On the other hand, according to theoretical speculation, Trp metabolites might take part in maintaining bone homeostasis by regulating immune cell activity and facilitating the differentiation of stem cells into OBs. However, compelling evidence that supports the association between bone homeostasis and immune system mediated by Trp remains absent. In summary, the intercellular coupling effects of Trp metabolites and their multifaceted roles within bone tissue underscore their pivotal importance in bone metabolism.

## Correlation between Trp Metabolism and Osteochondral Destruction Diseases

### Osteoarthritis

OA ranks among the most common degenerative joint disorders globally, manifesting through progressive cartilage degradation, synovial inflammatory responses, and pathological bone remodeling beneath the cartilage [115]. The pathogenesis of OA is complex and involves multiple factors, predominantly including obesity, occupational overuse of joints, middle-aged and elderly individuals, and certain genetic factors [116]. The primary clinical symptoms manifest as joint pain, stiffness, joint swelling, and restricted motor function. As a chronic progressive disorder, OA currently focuses on education, exercise, and weight loss, supplemented by nonsteroidal anti-inflammatory drugs (NSAIDs), corticosteroid injections, and several adjunctive medications, which has increasingly attracted research attention with the deficiency of a curative treatment [117]. Recent research has established a potential correlation between OA and Trp metabolism that may offer new insights into its pathophysiological mechanisms.

It has been well established that the pro-inflammatory Kyn-IDO pathway was activated in erosive hypertrophic osteoarthropathy (HOA), the severity of which was also negatively associated with Trp, IAld, and 3-OH-AA, but positively with 5-OH-Trp levels. Apart from the correlation with the symptoms of OA, its pain was also closely related to Trp metabolism. Serotonin and NAS levels exhibited a negative correlation with the number of tender joints. IAld level demonstrated a negative tender, while the levels of 3-OH-AA, 3-OH-Kyn, and 5-OH-Trp showed significant positive correlations with reported joint pain severity in patients [118]. The levels of TDO_2_, IL-1β, and TNF-α within the synovium of OA patients were dramatically boosted, among which elevated synovial TDO_2_ levels have been proven to be related to pro-inflammatory cytokines and OA condition [119]. The activated Kyn-AhR signaling pathway not only impaired the chondrogenic capacity of hUC-MSCs but also potentially undermined the therapeutic potential for cartilage protection in OA and related disorders [82].

Multiple 5-HTR have been described on the chondrocyte membrane. Serotonin stimulates phospholipase A2 in a dose-dependent manner, leading to reinforced activity of collagenase type II, which was probably implicated in the exacerbation of OA progression [85]. The SSRI, Flx, could promote gene expression of chondrogenic master regulator Sox9 and suppress the specific proteinase such as MMP-13, as well as function as an inhibitor targeting Wnt/β-catenin signaling, which not only decreased the total level of β-catenin but also accelerated binding of β-catenin with Axin1 and enhanced the phosphorylation dose dependently. Additionally, the down-regulation of Wnt/β-catenin signaling was further determined to efficiently contribute to alleviating OA progression in vivo [120].

It has been adequately summarized that melatonin served a protective role on OA cartilage, resulting in enhanced ECM production, attenuated chondrocyte apoptosis, impeded inflammatory mediators, and intervened matrix degradation, which was mechanistically involved in controlling the TGF-β, MAPK, or NF-κB signaling pathways [121]. From the perspective of gene expression, melatonin could restrain MMP-3, ADAMTS-4, MMP-13, inducible nitric oxide (NO) synthase (iNOS), and cyclooxygenase-2 (COX-2) and accelerate the expression of chondroprotective factor Col2 in chondrocytes [122–124]. In terms of mechanism, melatonin was instrumental in suppressing phosphorylation of phosphatidylinositol 3-kinase (PI3K)/AKT, p38, ERK, c-Jun N-terminal kinase (JNK), and MAPK, as well as activating NF-κB, exhibiting cytoprotective and anti-inflammatory properties in the oxidative stress-induced chondrocyte model. Recently, SIRT1 expression was identified to be prevalently decreased in OA cartilage of human and aged mice. Research reported that the alleviation effect of melatonin was reversed by SIRT1 small interfering RNA (siRNA) and sirtinol in the rabbit OA model, suggesting the position SIRT1 occupied [125]. Moreover, in 2022, melatonin has been confirmed to mitigate matrix degradation with enhanced SIRT1 expression via the NF-κB signaling pathway and preserve chondrocytes through activation of the TGF-β1/Smad2 pathway in IL-1β-induced rat chondrocytes [122]. In vitro, melatonin was also reported to assume a crucial role in maintaining mitochondrial functions and ECM synthesis, particularly related to SIRT1 expression, especially manifested as promoted ECM components, refined ATP creation, and decreased mitochondrial oxidative stress [126]. Meanwhile, the therapeutic impact of melatonin was concurrently supported by the expression and function of microRNAs. The melatonin-mediated activation of the miR-146a/NRF2/HO-1 axis acted as a paradigmatic instance to mitigate cartilage degeneration. Melatonin improved the protein levels of NRF2 through the suppression of miR-146a, thereby up-regulating antioxidant enzymes, primarily heme oxygenase 1 (HO-1), which promoted the anabolic metabolism of cartilage matrix in OA chondrocytes [127]. Remarkably, melatonin has also been proposed to have the potential to ameliorate OA progression by targeting chondrocyte mitochondrial oxidative stress [121]. It is widely recognized that the damaged regions in OA were commonly accompanied by iron accumulation. NOX4 could induce ferroptosis by targeting GRP78 downstream, diminishing the protective role of GPX4 and lowering its expression. Conversely, melatonin inhibited NOX4 expression in mitochondria to relieve mitochondrial dysfunction, efficiently hindering ferroptosis and easing OA [128]. Additionally, melatonin was implicated in the mitigation of oxidative injury by curbing cellular senescence and augmenting cartilage matrix biosynthesis, conferring protection to articular chondrocytes against H_2_O_2_-induced oxidative stress. Melatonin administration suppressed cytotoxicity induced by H_2_O_2_ and diminished the expression of inflammatory mediators including iNOS, COX-2, NO, and prostaglandin E2 (PGE2) in H_2_O_2_-stimulated human chondrocytes, reinstating the OA-compromised intracellular antioxidant defense mechanism within articular cartilage [129].

**Fig. 3.**
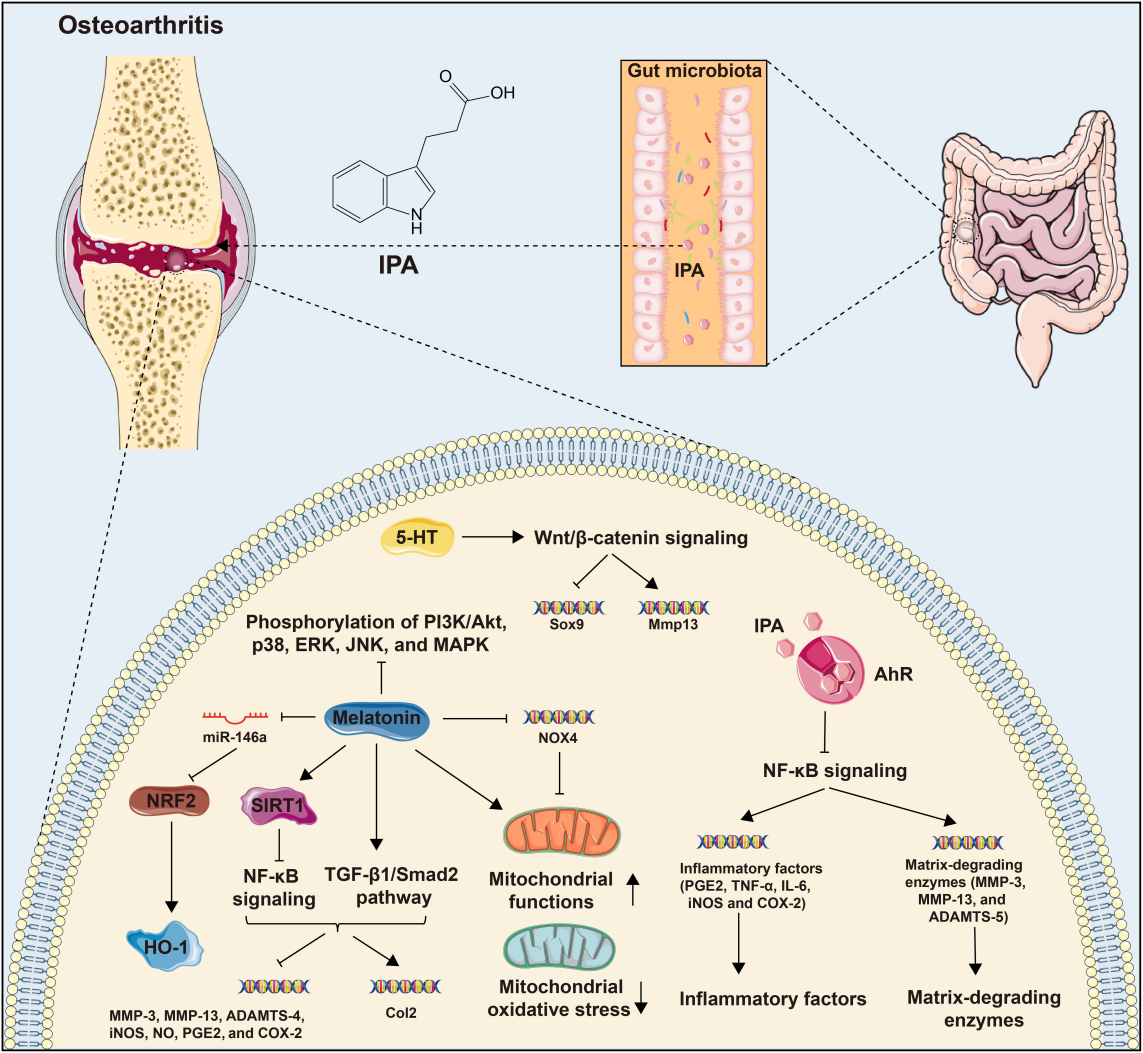
Interaction between Trp metabolites and OA. IPA targeted AhR to inhibit the expression of inflammatory factors (NO, PGE2, TNF-α, IL-6, iNOS, and COX-2) and matrix-degrading enzymes (MMP-3, MMP-13, and ADAMTS-5) by inactivating the NF-κB pathway. 5-HT activated Wnt/β-catenin signaling to inhibit gene expression of Sox9 and promote Mmp13. Melatonin inhibited the phosphorylation of PI3K/Akt, p38, ERK, JNK, and MAPK. Melatonin maintained mitochondrial functions, decreased mitochondrial oxidative stress, and inhibited NOX4 expression on mitochondria to mitigate ferroptosis. Melatonin significantly up-regulated HO-1 by enhancing the protein levels of NRF2 by suppressing miR-146a. Melatonin mitigated matrix degradation with enhanced SIRT1 expression by blocking the NF-κB signaling pathway and preserved chondrocytes through activation of the TGF-β1/Smad2 pathway, which inhibited the expression levels of MMP-3, MMP-13, ADAMTS-4, iNOS, NO, PGE2, and COX-2 and accelerated Col2 expression. [All original elements used in the schematic figures are acquired from Servier Medical Art (http://smart.servier.com/).]

IPA specifically acted on AhR to restrain the NF-κB signaling pathway, suppressing the expression of inflammatory factors (NO, PGE2, TNF-α, IL-6, iNOS, and COX-2) and matrix-degrading enzymes (MMP-3, MMP-13, and ADAMTS-5), as well as promoting anabolic markers including aggrecan and collagen-II, which perform to moderate OA progression in vivo [98]. Besides, cytochrome P450 of family 1, subfamily A, CyP1A1, and AhR expressions have been found to be positively correlated with the gravity of OA from patients. Further examination revealed that disturbance of the microbial biosynthesis of Trp and metabolites was associated with OA [130,131]. In the rat model of OA, antibiotic treatment resulted in decreased expression of AhR and CyP1A1 and lipopolysaccharide (LPS) levels while enhancing Col2A1 and SOX9 in chondrocytes, thereby alleviating the cartilage injury and synovitis, as well as diminishing the relative abundance of *Lactobacillus*. Intriguingly, extra Trp supplementation boosted intestinal microbiota-associated Trp metabolism, which might trigger the activation and generation of AhR, counteracting the influence of antibiotics and aggravating OA synovitis [132]. Especially reduced ILA levels in OA further elevated the risk of developing the condition [131] (Fig. 3).

Given the invaluable effect of Trp metabolism in the pathophysiology of OA, targeted therapeutic interventions are indispensable to mitigate symptoms and inhibit disease progression. Due to the potential therapeutic effects of melatonin in preventing OA via the management of circadian clock genes [133], reduction of chondrocyte apoptosis [134], anti-inflammatory properties [129], and free radical scavenging [135], the administration of melatonin and its bioactive derivatives presented a promising treatment option for OA. It has discovered intra-articular melatonin injection as an effective method of abating cartilage degeneration in rabbits afflicted with OA [129]. Expanding on this, a melatonin-laden drug delivery system (DDS) was developed to realize sustained release and intra-articular injection of DDS effectively lessened cartilage matrix degeneration in a posttraumatic rat OA model [126]. Tissue engineering was an effective approach for the remediation of cartilage and subchondral bone tissues. Recognizing the essential role of biomaterials in cartilage tissue engineering, various biomaterials, including fibrous scaffolds and hydrogels, have been gradually developed. Among various cartilage repair materials, hydrogel has gained considerable attention owing to the ECM-like microstructure and potential for drug delivery. Liu et al. [136] developed an innovative chemically modified biphasic hydrogel loaded with kartogenin and melatonin, which prompted both chondrogenic and osteogenic differentiation, achieving sustained release to repair osteochondral defects in a site-specific manner. An injectable gellan gum/lignocellulose nanofibril composite hydrogel, incorporating melatonin via forsterite nanoparticles, was also reported to enhance chondrocyte adhesion, proliferation, and ECM synthesis [137]. As previously discussed, distinct novel bioactive materials, including nanopolymers, cyclodextrins, liposomes, and hydrogels, have been extensively exploited [138,139], notably, within which the characteristic to load and transport melatonin ensuring sustained release and target articular cartilage are imperative to advance the clinical application of melatonin.

### Osteoporosis

OP represents a metabolic bone disease characterized by reduced BMD and increased skeletal fragility, primarily manifesting as trabecular bone loss and cortical thinning, which elevates the risk of fractures [140]. The phenomenon is more prevalent in females, particularly postmenopausal women, with epidemiological data indicating a rising incidence among the elderly population. Other risk factors include family history, hormonal imbalances, inadequate nutrition, a sedentary lifestyle, smoking, and chronic diseases [141]. OP can be categorized into 3 types comprising primary OP, such as postmenopausal OP (PMOP) and senile OP, secondary OP caused by other diseases or medication use, and idiopathic OP (IOP) occurring in young individuals with an unknown etiology. Clinical manifestations may encompass bone pain, height reduction, and vertebral compression fractures. Currently, there is no definitive cure, while available treatment options typically involve the use of anti-resorptive agents and anabolic agents, alongside increased dietary intake of calcium and vitamin D, as well as engagement in appropriate exercise [142].

Metabolomics analysis indicated a significant association between the oxidative metabolism of Trp and OP [13]. Kyn was increased alongside aging in murine bone marrow stromal cells [143]. Existing studies have shown that Kyn manifested a potential pathogenic role in age-induced bone loss, associated with suppression of BMSC proliferation, ALP activity, and osteogenic marker expression including OCN and Runx2 [144]. The Kyn-AhR axis was involved in suppressing starvation-induced autophagy and triggering senescence in BMSCs. Physiological levels of Kyn impaired autophagic flux in BMSCs, as indicated by decreased LC3B-II and autophagolysosomal generation, accompanied by a significant increase in p62 levels. Kyn treatment also triggered cellular senescence in BMSCs, as demonstrated by the up-regulation of characteristic senescence biomarkers such as senescence-associated β-galactosidase and p21, as well as enhanced nuclear H3K9me3 aggregation. Inhibition of AhR signaling reversed these effects, restoring autophagic flux and preventing the increase in senescence markers, presenting a potential therapeutic target to prevent or mitigate age-related bone loss and OP [145]. Lower baseline levels of 3-HAA were discovered in patients with OP; instead, significantly more AA and lipid peroxidation outcomes were tested compared with healthy controls [146]. Besides, it has been proved that high levels of certain Trp metabolites such as 3-HK and AA have a deleterious impact on bone homeostasis, reduced BMD, and increased fracture risk, while other metabolites including 3-HAA, XANA, PICA, QA, and NAD^+^ contribute to increasing BMD and lower risk of fracture [147]. A decrease in serum levels of muscle-derived KYNA, along with reduced KAT activity in the gastrocnemius muscle, was observed in a PMOP mouse model. The study demonstrated that either treadmill exercise, which up-regulated muscle KATs levels and increased serum KYNA concentration, or direct exogenous KYNA treatment could reduce NF-κB p65 phosphorylation by activating the Gpr35 receptor to inhibit NFATc1 expression in OCs while up-regulating Runx2 expression in OBs, ultimately mitigating bone mineral loss and microstructural deterioration in PMOP mice [148]. The products of the Kyn pathway may serve as promising targets for the advancement of new therapeutic approaches for OP. Based on the crucial role of Kyn-AhR that disrupted the equilibrium between bone resorption and bone formation in several mechanisms impelling age-associated bone loss, AhR antagonists such as CH-223191 and 3′,4′-dimethoxyflavone are attractive therapeutic approaches. In addition, both exercise intervention and exogenous KYNA treatment alleviated bone microstructure damage by restraining OC maturation and promoting OB viability, offering a novel therapeutic strategy for managing PMOP.

The opposite effects of the peripheral and central 5-HT signaling on bone have been sufficiently certified [51]. However, in clinical practice, 5-HT tended to exhibit an inhibitory function on OB activity and cause bone loss. As the first-line treatment drug for depression, SSRI was proposed to perform as a crucial risk element for OP [149]. Apparently, although 5-HT centrally curbed the sympathetic nervous system to relieve the negative adrenergic tone on OBs, the peripheral skeletal inefficiency induced by SSRI-mediated elevation of 5-HT seems to outweigh the skeletal benefits derived from the central enhancements [150]. Notably, current study reported that selectively suppressing biosynthesis of gut-derived serotonin (GDS) resulted in increased bone formation and effectively prevented or reversed OP in mice. LP533401, a small-molecule inhibitor of TpH_1_, significantly elevated bone formation markers and led to higher bone mass. Crucially, LP533401 had no impact on brain serotonin levels, which was essential given that GDS had opposing effects on bone metabolism [151,152]. At present, potentially serious adverse events of newer-generation antidepressant drugs encompassing OP and risk of fractures have not been resolved yet [153], while inhibitors of GDS synthesis such as LP533401 may represent a novel class of anabolic agents for the treatment of OP.

**Fig. 4.**
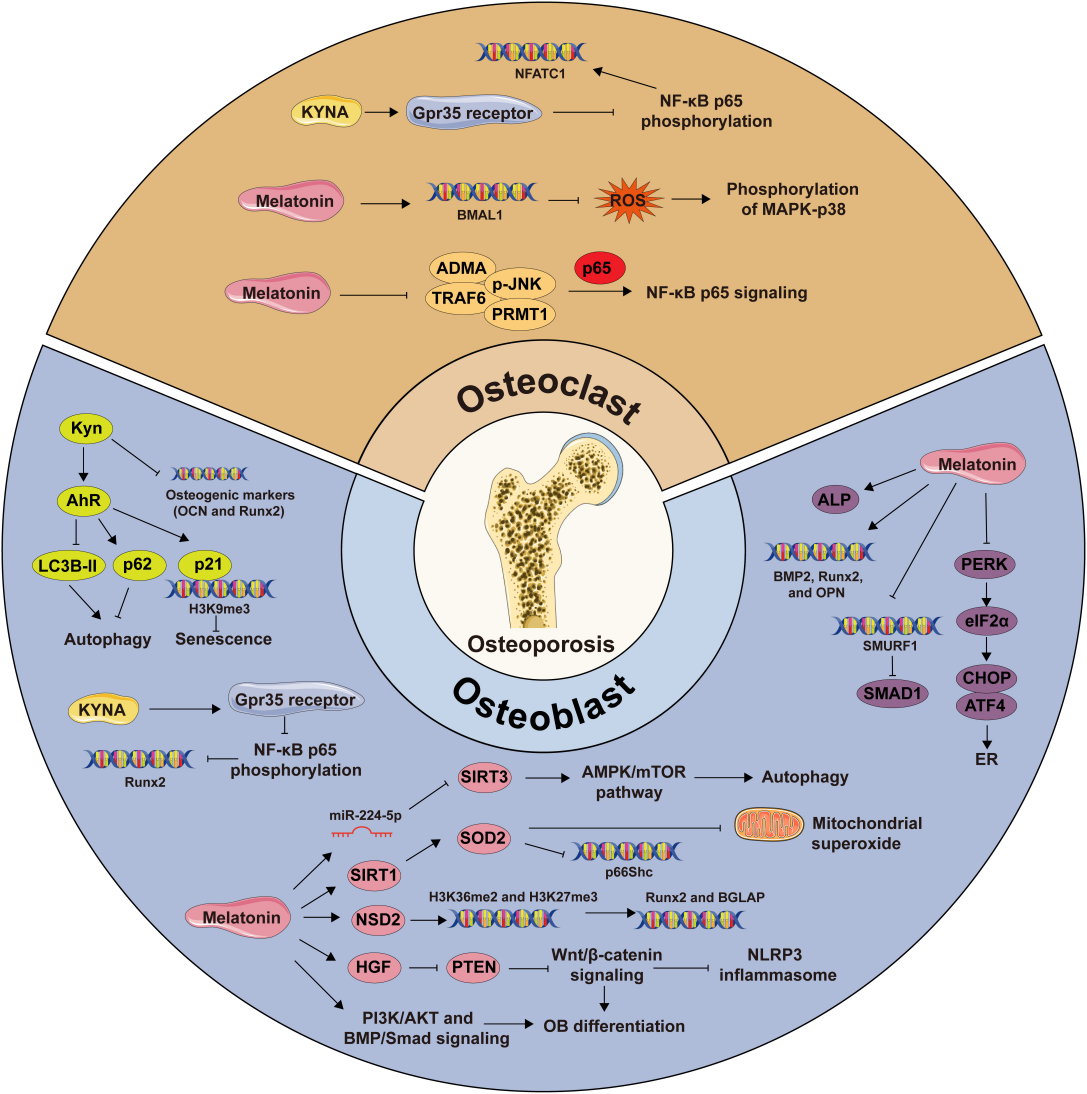
Interaction between Trp metabolites and OP. Osteoblastogenesis: Kyn inhibited osteogenic marker expression including OCN and Runx2. The kyn-AhR axis decreased LC3B-II and autophagolysosomal production, with increased p62 levels. Kyn also induced a senescent phenotype with up-regulated p21, as well as enhanced aggregation of nuclear H3K9me3. KYNA could reduce NF-κB p65 phosphorylation by activating the Gpr35 receptor to up-regulate Runx2 expression. Melatonin suppressed OB differentiation through activation of the PI3K/AKT and BMP/Smad signaling and restrained the autophagy-targeted miR-224-5p/SIRT3/AMPK/mTOR axis. Melatonin also enhanced osteogenic differentiation and delayed bone loss via the HGF/PTEN/Wnt/β-catenin axis and inhibited the activation of the NLRP3 inflammasome mediated by the Wnt/β-catenin signaling pathway. Melatonin decreased the level of mitochondrial superoxide by activating SIRT1 and its downstream SOD2. SIRT1 could also enhance SIRT3 and suppress p66Shc expression. Melatonin promoted NSD2 expression, leading to a rebalancing of H3K36me2 and H3K27me3 modifications to enhance the expressions of Runx2 and BGLAP. Melatonin reversed oxidation level and led to elevated ALP activity, and up-regulated expression of BMP2, Runx2, and OPN. Melatonin down-regulated SMURF1 expression, consequently reducing ubiquitination and degradation of SMAD1 protein. Melatonin significantly relieved ERS by inhibiting the cascade of the PERK–eIF2α–ATF4–CHOP signaling axis. Osteoclastogenesis: KYNA could reduce NF-κB p65 phosphorylation by activating the Gpr35 receptor to inhibit NFATc1 expression. Melatonin increased BMAL1 expression to inhibit the activation of ROS and phosphorylation of MAPK-p38. It also obstructed osteoclastogenesis by inhibiting PRMT1 and ADMA expression, as well as TRAF6 and the phosphorylation of JNK. Melatonin also restrained the transcriptional activity of NF-κB by disturbing the binding of PRMT1 and NF-κB subunit p65. [All original elements used in the schematic figures are acquired from Servier Medical Art (http://smart.servier.com/).]

Melatonin exhibits potential therapeutic effects across various types of OP by promoting osteogenesis, inhibiting osteoclastic resorption, attenuating oxidative damage, and modulating immune system regulation. Melatonin mitigated glucocorticoid (GC)-induced suppression of OB differentiation through activation of the PI3K/AKT and bone morphogenetic protein (BMP)/Smad signaling pathways in MC3T3-E1 cells [154]. It also targeted the miR-224-5p/sirtuin 3 (SIRT3)/AMPK/mammalian target of rapamycin (mTOR) axis to alleviate OP progress and suppressed autophagy in GC-treated hBMSCs. In vitro, melatonin decreased miR-224-5p expression to up-regulate SIRT3, which was involved in the inactivation of the AMPK pathway, thereby rescuing the GC-induced OP and autophagy inhibition [155]. PMOP accounted for approximately two-thirds of all cases, representing the most prevalent form of the disease. Melatonin was discovered to enhance bone density and improve bone metabolism in normal, perimenopausal, and postmenopausal osteoporotic rats by promoting osteogenic differentiation in BMSCs [156]. Melatonin potentially enhanced BMSC proliferation and osteogenic differentiation and delayed bone loss through the hepatocyte growth factor (HGF)/phosphatase and tensin homolog deleted on chromosome ten (PTEN)/Wnt/β-catenin axis, which reversed the down-regulation of HGF to diminish PTEN expression, leading to the activated Wnt/β-catenin pathway both in vitro and in vivo [157]. Melatonin also repressed the activation of the NLRP3 inflammasome mediated by the Wnt/β-catenin signaling pathway to mitigate estrogen deficiency-induced OP [158]. The SIRT1–superoxide dismutase 2 (SOD2) axis has been underscored in melatonin-enhanced mitochondrial energy metabolism in OVX-BMSCs. Melatonin decreased the level of mitochondrial superoxide by activating SIRT1 and its downstream antioxidant enzymes, particularly SOD2 [159]. Concurrently, melatonin could enhance osteoporotic bone repair by facilitating BMSC-driven angiogenesis and osteogenesis–angiogenesis coupling in OVX rats, as evidenced by elevated expression of osteogenic markers such as ALP, OCN, Runx2, and Osterix, alongside angiogenic markers such as vascular endothelial growth factor (VEGF), angiopoietin-2, and angiopoietin-4. Moreover, it fortified the bone strength of the tibia defect, as indicated by augmented ultimate load and stiffness demonstrated through the 3-point bending test [160]. While promoting osteogenesis, melatonin also had an inhibitory effect on osteoclastogenesis in estrogen deficiency-induced OP. Melatonin could accelerate cell apoptosis through BMAL1/ROS/MAPK-p38 in RAW264.7 cells, specifically increasing BMAL1 expression to block the activation of ROS and phosphorylation of MAPK-p38 [161]. Besides, the anti-osteoclastogenic effect of melatonin was manifested by a cascade of RANKL-induced tumor necrosis factor receptor-associated factor 6 (TRAF6), JNK, protein arginine methyltransferase 1 (PRMT1), and NF-κB signaling inhibition. More specifically, melatonin treatment efficiently obstructed osteoclastogenesis by inhibiting PRMT1 and asymmetric dimethylarginine (ADMA) expression, as well as suppressed RANKL-induced TRAF6 and the phosphorylation of JNK in the MT-independent pathway. Melatonin also restrained the transcriptional activity of NF-κB by disturbing the binding of PRMT1 and NF-κB subunit p65 in BMMs [162]. In clinical settings, the gravity of senile osteoporosis (SOP) was inversely correlated with melatonin levels in the bone marrow. Melatonin promoted the expression of the histone methyltransferase NSD2 through MT_1/2_-mediated signaling pathways, leading to a rebalancing of H3K36me2 and H3K27me3 modifications to enhance chromatin accessibility for osteogenic genes such as Runx2 and BGLAP, thereby promoting osteogenesis of bone marrow stromal cells in vitro and mitigating the progression of OP in aging mice [59]. Melatonin also showed promising potential in addressing inflammation-induced OP, primarily owing to its multifaceted functions in modulating bone homeostasis and inflammatory responses. Retinoic acid-induced OP model mice manifested by developed OCs and restrained osteogenesis due to the increasing oxidative stress levels in the RAW264.7 and MC3T3-E1 cells, which could be reversed by melatonin in enhancing bone formation, repairing the trabecular microstructure, and alleviating bone loss [163]. In H₂O₂-exposed MC3T3-E1 cells, melatonin effectively mitigated oxidative damage and markedly enhanced osteogenic differentiation through the activation of SIRT1, which in turn regulated SIRT3 activity and inhibited p66Shc expression. Melatonin treatment led to elevated ALP activity, enhanced mineralization capacity, and up-regulated expression of osteogenic markers, including BMP2, Runx2, and OPN. Furthermore, it resulted in decreased intracellular ROS levels, mitochondrial stabilization, reduced malondialdehyde levels, increased SOD activity, and a significant reduction in apoptosis [164]. Melatonin was demonstrated to rescue TNF-α-induced suppression of osteogenesis in hMSCs by modulating the interaction between SMURF1 and SMAD1. Specifically, the crosstalk between melatonin signaling and TNF-α signaling pathways was observed to down-regulate SMURF1 expression, consequently reducing SMURF1-mediated ubiquitination and degradation of SMAD1 protein, resulting in the stabilization of BMP-SMAD1 signaling activity and restoration of osteogenesis compromised by TNF-α [165]. Except for its role in inflammation-induced OP, melatonin may offer therapeutic benefits in managing OP associated with diabetes, given the overlapping pathophysiological mechanisms involving impaired bone metabolism and chronic inflammation. Hyperglycemia diminished cellular viability and promoted apoptosis in osteoblastic cell lines. High glucose triggered ERS by enhancing calcium flux and up-regulating the ER chaperone, a binding immunoglobulin protein (BiP). Meanwhile, it induced the post-translational activation of eukaryotic initiation factor 2α (eIF2α), the downstream of PKR-like ER kinase (PERK), which resulted in the activation of activating transcription factor 4 (ATF4) and the up-regulation of CHOP, which performed as ER stress-mediated apoptosis regulator, along with its downstream effectors DNAJC3, HYOU1, and CALR. Melatonin administration has been shown to significantly relieve hyperglycemia-induced alterations in cellular growth, apoptosis, and calcium influx by inhibiting the cascade of the PERK–eIF2α–ATF4–CHOP signaling axis [166]. IOP has been increasingly linked to genetic factors, with emerging research identifying potential molecular targets. With whole-exome sequencing analyses across various IOP cohorts, several variants in the MT_1A_ gene have been certified that may have pathogenic consequences, which supported the notion that mutations in MT_1A_ contributed to the genetic basis of IOP and highlighted the rs374152717 variant as a loss-of-function allele promoting senescence to affect bone turnover in OBs [167] (Fig. 4).

Recent studies suggested that melatonin could be a potential therapeutic agent for OP-related bone metabolic diseases, offering new insights into its potential as a treatment. Notably, daytime administration of melatonin demonstrated superior efficacy in preventing bone loss compared to nighttime administration in OVX rats, as evidenced by denser bone microarchitecture. Additionally, biomechanical and molecular analyses revealed that daytime treatment enhanced bone strength, increased bone formation markers, reduced bone resorption markers, and improved cell viability and resistance to oxidative stress at lower melatonin doses, ultimately reducing apoptosis more effectively [168]. Besides, multiple investigations also provided evidence for the potential application of integrating melatonin with bioactive materials in osteoporotic bone defects. Melatonin-loaded silk fibroin scaffolds (SF@MT) were developed for localized and sustained release, and implantation in OVX rats with femur defects demonstrated partial restoration of mitochondrial energy metabolism and osteogenic differentiation in OVX-BMSCs, which highlighted the potential of localized melatonin delivery via bone implants to treat osteoporotic bone defects by reestablishing mitochondrial redox homeostasis [159]. All the above provided a safe and rational strategy for the intervention of various OP.

The underlying intestinal microbiota-associated Trp metabolism–OP connection has been established. OP disrupted intestinal Trp metabolism and reduced the production of gut microbiota-derived melatonin, whereas melatonin supplementation alleviated related symptoms and rectified gut microbiota dysbiosis, enhancing microbial diversity, increasing the relative abundance of key probiotics such as *Allobaculum* and *Parasutterella*, and improving metabolic functions. Moreover, melatonin elevated short-chain fatty acid production while reducing trimethylamine N-oxide metabolites, as well as modulated the M1/M2 macrophage balance, lowered serum pro-inflammatory cytokine levels, and restored gut barrier integrity [169]. Supplementation with Trp metabolites, specifically IAA and IPA, could restore intestinal barrier integrity probably through the Wnt/β-catenin signaling pathway in AhR-dependent mechanisms, significantly mitigating bone loss in the OVX mice model. Additionally, M2 macrophages induced by IAA and IPA secreted substantial IL-10, extending from the intestinal lamina propria to the bone marrow, thereby promoting osteoblastogenesis while inhibiting osteoclastogenesis both in vivo and in vitro [170]. Microbial Trp metabolites show potential as therapeutic agents for OP by modulating the gut–bone axis. Ursolic acid derivatives were successfully designed under the guidance of docking technique, with compound 9a identified as a strong Tph-1 binder via surface plasmon resonance (SPR) analysis. Compound 9a inhibited Tph-1 expression, lowering serotonin levels in the serum and gut while sparing brain serotonin. Furthermore, oral administration of 9a elevated N-terminal propeptide of procollagen type 1 (P1NP), a marker of bone formation, and enhanced bone microarchitecture [171].

### Rheumatoid arthritis

Rheumatoid arthritis (RA) is a long-term autoimmune condition primarily affecting the synovial joints, characterized by widespread inflammation and resulting in gradual joint damage and functional limitations [172]. Pathologically, RA is marked by the infiltration of immune cells, predominantly T lymphocytes and macrophages, into the synovial membrane, resulting in synovitis and the development of pannus, which invades and erodes adjacent cartilage and bone. The disease exhibits a global prevalence of approximately 0.5% to 1%, with a notable female predominance and an onset typically occurring between the ages of 30 and 50. The risk factors include genetic predisposition, particularly HLA-DRB1 alleles, environmental triggers like smoking and silica exposure, hormonal influences, and lifestyle factors such as obesity and diet [173]. The representative clinical manifestation is symmetrical polyarthritis, which often involves the finger and toe joints, accompanied by prolonged morning stiffness and extra-articular symptoms including rheumatoid nodules and lung-related manifestations. Current management involves a multidisciplinary approach featuring anti-rheumatic drugs that can modify the disease course such as methotrexate, biological agents targeting specific inflammatory pathways, NSAIDs, and GCs for symptom relief [174]. Aberrant Trp metabolism disrupts the immune–bone crosstalk in RA by dysregulating key cellular players, including T helper 17 (Th17)/Treg balance, promoting macrophages and fibroblasts, while altering critical factors such as AhR signaling, pro-inflammatory cytokines, and osteoclastogenesis [175]. TDO2 expression was strongly increased in synovial tissue and fibroblast-like synoviocytes, which may contribute to synovial inflammation and joint destruction during arthritis, leading to elevated proliferation, secretion, migration, and invasion [176]. However, based on the involvement of activated synovial fibroblasts and infiltrating T lymphocytes forming a self-sustaining inflammatory circuit in joint destruction, research focused on immune cells reported that transplantation of TDO2-overexpressing dendritic cells significantly alleviated collagen-induced arthritis (CIA) in mice by rebalancing Th17 and Treg cell populations [177]. Moreover, under the condition of pathogenesis, hypoxia disrupts synovial fibroblast–Th cell interactions by weakening proliferation control and boosting IL-17A production in RA [178].

Current research proposed that the intricate interplay among Trp, its metabolites, and intestinal flora emerged as a crucial factor in the pathophysiological process and inflammatory trajectory of RA. It was reported that the Kyn and indole pathways of Trp metabolism have been implicated more in RA pathogenesis [179,180]. Patients with RA exhibited significant fluctuations in Kyn metabolite levels when compared to healthy individuals. Specifically, serum concentrations of KYNA, XANA, and indole derivatives were observed to be reduced, while QA levels were elevated. The metabolic alterations were positively correlated with the disease severity assessed by circulating biomarkers and disease activity indices, and inversely related to life quality [181]. Moreover, from the perspective of the local inflammatory site, KYNA, an endogenous metabolite of Trp, has been identified in synovial fluid of RA, which played an inhibitory role in synoviocyte proliferation, as well as reinforced antiproliferative function of celecoxib and nimesulide in subthreshold concentration of 0.3 mM [182]. However, quinaldic acid (QUDA), which was capable of suppressing both the growth and movement of synovial cells according to the dosage, presented as a state of local deficiency in RA patients, resulting in the loss of synovial hyperplasia inhibition [183]. RA patients also exhibited enhanced IDO activity [184]. In lymph nodes, Trp concentration decreased notably during arthritis progression, and elevated Kyn levels suggested IDO activation. A subsequent examination of the metabolites of Kyn during the remission phase of arthritis revealed a substantial accumulation of AA and 3-HAA [185,186].

Trp metabolites of the Kyn pathway might be regarded as a novel treatment approach in RA. QA was found to enhance the growth of human fibroblast-like synovial cells and activate mitochondrial respiration and sugar metabolism. Additionally, the whole-body delivery of aminoadipate aminotransferase, which catalyzed the synthesis of XANA and KYNA, showed a safeguarding effect. Changes in Trp metabolism have also been verified to play a positive role in the development of RA in preclinical and clinical settings [181]. A deeper understanding of the specific molecular targets of individual Kyn metabolites is likely to be crucial [187]. Both Kyn and KYNA serve as activators of AhR, suggesting that the development of more selective AhR agonists could offer clinical benefits [188,189].

Serotonin levels in the serum were found to be increased not only at the onset of RA but also preceding its development [190]. Variations in the 5-HTR gene influence immune reactions in RA. The expression of 5-HTR_2A_ was significantly decreased in individuals with RA, which may be attributed to either a predisposition to the disease or a consequence of its progression [191]. Further research proposed that the methylation levels of HTR_2A_ in circulation are linked to inflammatory responses and disease severity of RA. Meanwhile, the hypermethylation within the promoter area of the HTR_2A_ gene also implies a potential role in the clinical diagnosis [192]. Additionally, the association of RA with a specific 5-HTR haplotype has functional implications, as it alters the immunological characteristics of T cells and monocytes [193]. In TpH_1_ knockout mice suffering from arthritis, there was a significant increase in OC differentiation and bone resorption. In the paws, the levels of IL-17 were elevated. Moreover, there was a rise in the number of Th17 lymphocytes in the draining lymph nodes, whereas the activity of Treg cells was inhibited.

Interestingly, when serotonin and specific agonists for the 5-HTR_2A_ and 5-HTR_2B_ receptors were applied outside the living body (ex vivo), they effectively restored the secretion of IL-17 from splenocytes and the differentiation of Th17 cells. This finding highlighted the regulatory role of serotonin in arthritis by balancing the ratio between Th17 and Treg cells and having an impact on OC formation [194].

Except for 5-HT modulating immune responses in arthritis, the function of melatonin, a hormone derived from 5-HT, was constantly explored in bone homeostasis. While melatonin has demonstrated positive effects in various animal models and clinical trials for inflammatory autoimmune conditions, its impact in the context of RA remains a subject of debate [195]. The first research about a positive genetic association between MT_1B_ polymorphism (rs 1562444) and rheumatoid factor in RA was reported in Korea [196]. The current body of literature offers conflicting views regarding the impacts of melatonin on patients with RA. Certain research findings have suggested that melatonin might exacerbate disease activity by intensifying proinflammatory responses with preclinical and clinical evidence [197]. Melatonin level was relatively high in synovial fluid of patients with RA, and binding sites for melatonin were identified in synovial macrophages [198]. Additionally, synovial macrophages cultured from patients with RA exhibit heightened production of proinflammatory cytokines in response to melatonin stimulation [199,200]. Hansson et al. [201] found that continuous darkness aggravated CIA in DBA/1 mice. In contrast, continuous light had a weaker influence on the concentration of serum anti-collagen antibodies. This demonstrated that disturbing the rhythms of the pineal hormone melatonin to reach a high physiological level of this indoleamine could activate the immune system, leading to the worsening of autoimmune collagen II arthritis. On the other hand, eliminating pineal melatonin production had a protective effect [202]. Furthermore, considering the association between RA and the immune system, it was hypothesized that the melatonin secreted by the pineal gland might enhance the activation of T cells, thereby worsening the progression of CIA [203]. Beyond the impact of lighting conditions, disturbances in the body’s internal biological clock played a pivotal role in the development of RA [204]. Melatonin influenced RA pathogenesis by modulating the transcription of clock-related genes, for example, Cry1 [205]. Suppression of melatonin on Cry1 gene expression led to enhanced cAMP levels and activation of PKA and NF-κB, thereby exacerbating CIA in rat models [206,207]. Several other research projects have disclosed that melatonin possesses remarkable anti-inflammatory and immunoregulatory properties in preclinical models of arthritis. Both the concentration and duration of melatonin administration significantly influence its therapeutic effects. A randomized controlled trial suggested that daily 10-mg dose of melatonin in arthritis patients exhibited a gradual antioxidant effect and elevated certain inflammatory markers but inconsistent alterations in proinflammatory cytokine levels or improvements in clinical symptoms [208]. Melatonin mitigated inflammation and modulated thymocyte function in adjuvant-induced arthritis, with its effects linked to the G protein–adenyl cyclase–cAMP pathway and Met-enkephalin (Met-Enk) release. Specifically, melatonin strikingly reversed the suppression of thymocyte propagation resulted from reduced level of Met-Enk in AA rats, as well as suppressed inflammation, enhanced IL-2 secretion, and reduced forskolin-induced cAMP levels. Moreover, the concentration gradient analysis revealed that melatonin stimulated lymphocyte proliferation in normal rats only at higher doses, whereas comparable effects were achieved in AA rats at significantly lower concentrations [209]. In vitro, administering melatonin notably decreased the synthesis of TNF-α and IL-1β in synovial fibroblasts from human RA patients. This effect was achieved by suppressing the PI3K/AKT, ERK, and NF-κB signaling pathways and up-regulating the expression of miR-3150a-3p. Studies further confirmed that its anti-inflammatory effects were mediated by MT_1_, which curbed inflammatory cytokine secretion and lessened cartilage breakdown and bone resorption associated with CIA [89]. Melatonin demonstrated a complex dual function, acting simultaneously as a factor promoting inflammation and an antioxidative agent in rat CIA models. Jiménez-Caliani et al. [210] discovered that melatonin treatment in the dose of 30 μg through the back of the rat tail increased the levels of anti-collagen antibodies, along with IL-1β and IL-6, in the serum and joints of arthritic rats. Meanwhile, it decreased oxidative stress indicators such as nitrite, nitrate, and lipid peroxidation in the serum, yet exerted no such effect in the joints. This bidirectional regulation was influenced by multiple factors, including dosage, timing of administration, local microenvironment including gut microbiota-mediated metabolic variations, and individual immune status such as Th17/Treg balance. Additionally, the differential expression of its receptors MT_1_/MT_2_ in various immune cells further contributed to its diverse roles in RA, leading to either protective or disease-promoting effects.

**Fig. 5.**
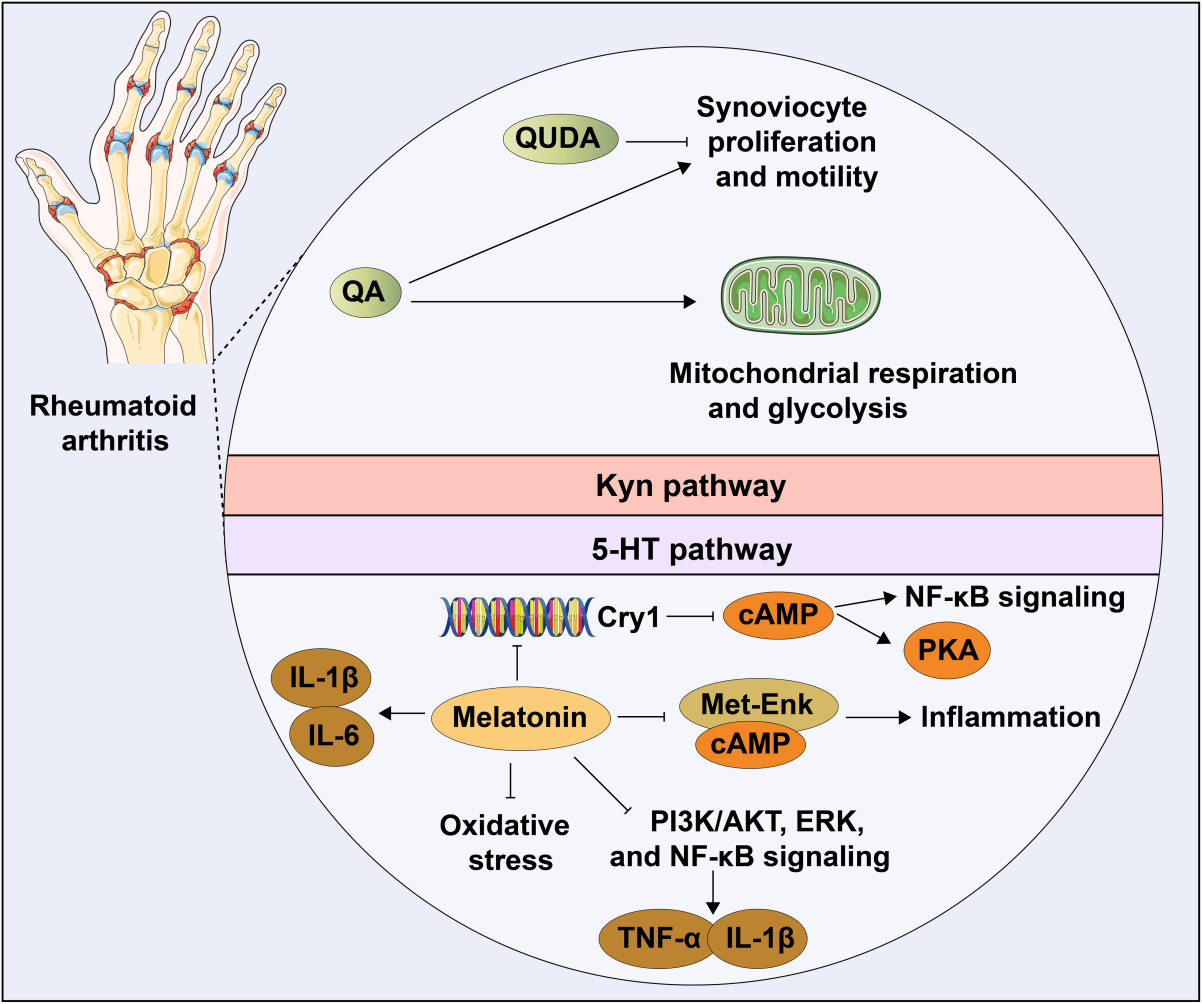
Interaction between Trp metabolites and RA. In the Kyn pathway, QUDA can restrain both the proliferation and motility of synoviocytes. QA can promote the proliferation of human fibroblast-like synoviocytes and stimulate mitochondrial respiration and glycolysis. In the 5-HT pathway, melatonin inhibited Cry1 expression, leading to enhanced cAMP levels and activation of PKA and NF-κB. Melatonin mitigated inflammation with the G protein–adenyl cyclase–cAMP pathway and Met-Enk inhibition. Melatonin significantly reduced the production of TNF-α and IL-1β by inhibiting PI3K/AKT, ERK, and NF-κB pathways. Besides, melatonin elevated IL-1β and IL-6 levels and reduced oxidative stress. [All original elements used in the schematic figures are acquired from Servier Medical Art (http://smart.servier.com/).]

Regarding indole derivatives, empirical data on human subjects are limited. Nevertheless, the importance of Trp metabolites derived from the microbiota is underscored by extensive research showing that these compounds, acting as agonists of AhR, perform an important function in managing immune disorders [211], which achieved relief by modulating the microbial community, preserving the intestinal barrier, balancing immunity, and bone impairment in individuals suffering from RA, efficiently bridging the connection between the gut and joints [212]. Emerging research focused on their function to ameliorate RA via the gut–bone crosstalk, which was considered a therapeutic target [213]. Sinomenine (SIN) has been identified to mitigate the symptoms of CIA by restoring the equilibrium of gut microbiota, particularly by increasing *Lactobacillus* levels, and elevating microbial Trp metabolites such as indole-3-acrylic acid (IA), IPA, and IAA, which activated AhR, modulating the Th17/Treg balance in CIA rats. Through mono-colonization, 2 beneficial *Lactobacillus* species with anti-CIA properties, *L. paracasei* and *L. casei*, were enriched, which successfully mitigated arthritis, implying the prospective therapeutic function of SIN [214]. Oral liquid of *Saussurea involucrata* efficiently mitigated symptoms of RA, including joint pain, swelling, and morning stiffness. This effect may be achieved by regulating the richness of the gut microbiome of the *Lactobacillus*, *Romboutsia*, *Bacteroides*, and *Alloprevotella* genera. Such regulation enhanced the tricarboxylic acid (TCA) cycle, phenylalanine metabolism, and the biosynthesis of phenylalanine, tyrosine, and Trp, along with pathways related to glyoxylate and dicarboxylate metabolism [215]. More effective or adjunctive therapeutic options are pressingly needed (Fig. 5).

### Ankylosing spondylitis

Ankylosing spondylitis (AS), a type of arthritis with a progressive inflammatory nature, mainly affects the axial skeleton and is characterized by sacroiliitis, also marked by the development of syndesmophytes and the inflammation of enthesitis, primarily at the entheseal sites where tendons, ligaments, or joint capsules attach to bone, potentially resulting in spinal fusion over time. It manifests as inflammation, chronic lumbago, restricted mobility, and decreased life quality [216]. The prevalence ranges from 0.1% to 2% across various populations, with a higher incidence observed in young and middle-aged males [217]. The etiology of AS remains not entirely elucidated, but it is widely accepted that a combination of genetic predisposition, environmental influences, and immune responses significantly contributes to its development [218]. Trp metabolism is reported to influence AS through multiple pathways, including immune modulation, inflammation, gut microbiome interactions, and oxidative stress [219,220].

Various research indicated that pro-inflammatory cytokines, like interferon-γ (IFN-γ), TNF-α, IL-1β, and IL-6, performed a pivotal function in the progression of AS. Such pro-inflammatory cytokines were capable of modulating the functioning of enzymes within the Kyn pathway, which in turn could induce metabolic alterations that influence both inflammation and immune function [221]. More specifically, the levels of serum Trp, KYNA, and 3-HK were significantly decreased among individuals suffering from AS, while Kyn, QA, C-reactive protein (CRP), erythrocyte sedimentation rate (ESR), and IL-6 levels were elevated [222]. Kyn treatment during differentiation significantly boosted OPG and OCN expressions in AS osteoprogenitors, enhancing bone mineralization and suppressing RANKL-mediated OC differentiation, which suggested that irregular Kyn levels might serve as a regulatory link between OC and OB functions, possibly playing a role in the characteristic bone pathology of AS [108].

The concentrations of serotonin in patients diagnosed with AS were significantly diminished when compared to those in healthy subjects, with an even more pronounced decrease observed in those undergoing TNF-α blocker therapy. An inverse correlation was detected between the levels of serotonin and the activation of phosphorylated cAMP response element binding protein (pCREB) in OB-like Saos-2 cells, demonstrating a potential role for serotonin in the process of osteogenesis associated with AS [223]. Conversely, melatonin levels were elevated in AS patients with higher levels of the spinal bone bridge. Melatonin has been recognized as a risk factor for spinal bone formation through multiple linear regression analysis and shown positive correlations with OCN and IL-1β in AS, suggesting its critical role in pathological bone formation [224]. The clinical assessment of AS was conducted using the Bath AS Disease Activity Index (BASDAI), and functional impairment was evaluated with the Bath AS Functional Index (BASFI). Melatonin levels demonstrated a positive correlation with BASDAI, BASFI, the period of morning stiffness, and CRP concentration, yet no correlation was found with ESR, which implied that it may function as a signpost for gauging disease activity in those diagnosed with AS [225,226].

**Fig. 6.**
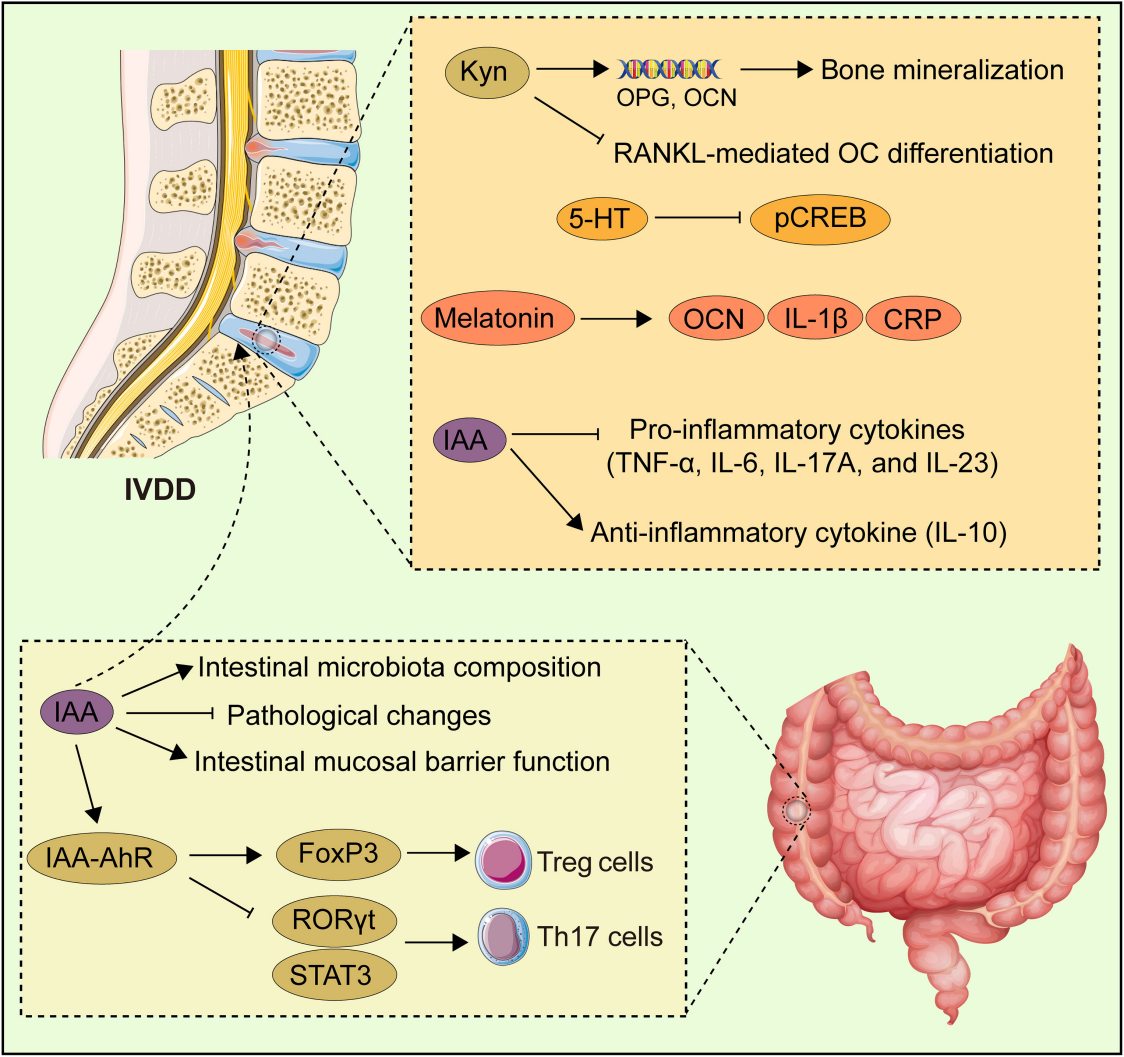
Interaction between Trp metabolites and AS. Kyn significantly boosted OPG and OCN expressions by enhancing bone mineralization and suppressing RANKL-mediated OC differentiation. There was an inverse relationship between 5-HT levels and pCREB activation. Melatonin showed a positive correlation with OCN and IL-1β expression, as well as BASDAI, BASFI, the duration of morning stiffness, and CRP levels. IAA derived from gut microbiota down-regulated cytokine levels including TNF-α, IL-6, IL-17A, and IL-23, as well as improved the generation of anti-inflammatory cytokine IL-10. IAA could also enhance the intestinal barrier, relieve pathological changes, and reshape the gut microbiota. IAA stimulated the AhR pathway, up-regulating FoxP3 and increased Treg cells, down-regulating RORγt and STAT3, and decreasing Th17 cells. [All original elements used in the schematic figures are acquired from Servier Medical Art (http://smart.servier.com/).]

Furthermore, AS was reported to be associated with intestinal microbiota imbalances. IAA, a microbial metabolite of Try in the indole pathway, has shown potential in managing AS by modulating gut homeostasis and dampening inflammation. IAA could reduce AS incidence and severity, regulate cytokine production including TNF-α, IL-6, IL-17A, and IL-23, as well as improve the generation of anti-inflammatory cytokine IL-10. It was revealed that IAA activated the AhR pathway. This activation led to an up-regulation of the transcription factor forkhead box protein P3 (FoxP3), subsequently increasing Treg cells. Concurrently, IAA caused a down-regulation of the transcription factors retinoic acid receptor-related orphan receptor γt (RORγt) and signal transducer and activator of transcription 3 (STAT3), which in turn led to a decrease in Th17 cells. In addition to elevating the abundances of *Bifidobacterium pseudolongum* and *Mucispirillum schaedleri*, IAA was also capable of strengthening the intestinal barrier and restructuring the gut microbiota. Specifically, it led to an augmentation of *Bacteroides* and a reduction in *Proteobacteria* and *Firmicutes*. The multifaceted effects of IAA suggested that it could be a new therapeutic approach for AS, targeting intestinal microbiota, immune response, and inflammation [227] (Fig. 6).

### Other skeletal anomalies

Intervertebral disk degeneration (IVDD) is responsible for the occurrence of low back pain and gives rise to modifications in the spinal structure, which include changes in the shape, arrangement, and integrity of the spinal components. The Trp2 allele of COL9A2 was proven as an age-dependent risk factor that influenced the degree of disc degeneration among younger patients with symptomatic lumbar herniated nucleus pulposus [228]. The first study to explore melatonin’s role in IVDD repair in a rat model was reported in 2006. Melatonin treatment has been shown to reverse the reduction of trabecular width, increase TGF-β1 expression, and improve disk appearance, suggesting a potential recovery process activation in IVDD tissue [229].

Scoliosis represents a complicated spinal malformation that is distinguished by a lateral curvature of the vertebral column, which can cause physical discomfort and impaired mobility. Pinealectomized-induced melatonin deficiency exhibited reduced OB proliferation and led to scoliosis, while melatonin administration could reverse the development of scoliosis and bone loss [230].

Small cell lung cancer (SCLC) is an extremely invasive type of cancer, making up roughly 15% of all instances of lung cancer [231]. A significant proportion of SCLC patients, ranging from 50 to 60%, experience the development of osteolytic bone metastases, which severely diminishes their quality of life. Kyn was discovered to not only activate AhR associated with the advancement and spread of SCLC but also enhance osteoclastogenesis. The fundamental mechanism entailed that treating with Kyn enhanced the migratory and clonogenic abilities of SCLC cell lines through the activation of the ERK/AKT oncogenic signaling pathway; meanwhile, Kyn promoted OB-mediated OC differentiation via RANKL. By restraining AhR with clofazimine (CLF), there was a significant decline in the proliferation of SCLC cells induced by Kyn, along with an elevation in apoptosis and an arrest of the cell cycle in the G_2_/M phase. CLF treatment also diminished RANKL expression to alleviate bone resorption. All these indicate the instrumental function in promoting SCLC therapies that are effective against bone metastasis [232]. Additionally, in bone metastasis, leucyl-Trp was identified as a biomarker in breast cancer detection [233]. As for primary tumor osteosarcoma, elevated IDO expression was listed as an independent risk factor, significantly correlating with reduced metastasis-free and overall survival. IDO-mediated immune tolerance may influence osteosarcoma tumorigenesis and affect clinical outcomes, providing a potential target for immunotherapy in osteosarcoma treatment [234] (Table 2).

**Table 2.** Relevant functions of Trp metabolites in the pathophysiology of osteochondral destruction diseases

Diseases	Metabolites	Functions	References
OA	5-HT	5-HT inhibition promoted gene expression of chondrogenic master regulator Sox9, suppressed the specific proteinase such as Mmp13, and inhibited Wnt/β-catenin signaling.	[120]
	Melatonin	Mitigated matrix degradation with enhanced SIRT1 expression via NF-κB signaling pathway and preserved chondrocytes through activation of the TGF-β1/Smad2 pathway, inhibited the expression levels of MMP-3, MMP-13, ADAMTS-4, iNOS, and COX-2, and accelerated Col2 expression.	[122,123,129]
		Suppressed phosphorylation of PI3K/Akt, p38, ERK, JNK, and MAPK, as well as activated NF-κB, exhibiting cytoprotective and anti-inflammatory properties in the oxidative stress-induced chondrocyte model.	[123]
		Maintained mitochondrial functions, refined ATP creation, and decreased mitochondrial oxidative stress.	[126]
		Mitigated cartilage degeneration through the miR-146a/NRF2/HO-1 axis.	[127]
		Inhibited NOX4 expression in mitochondria to hinder ferroptosis and ease OA.	[128]
	IPA	Restrained the NF-κB signaling pathway, suppressed inflammatory factors and matrix-degrading enzymes, and promoted anabolic markers including aggrecan and collagen-II.	[98]
OP	Kyn	Suppressed BMSC proliferation, alkaline phosphatase activity, and osteogenic marker expression.	[144]
		Involved in suppressing starvation-induced autophagy and triggering senescence in BMSCs.	[145]
	KYNA	Reduced NF-κB p65 phosphorylation by activating the Gpr35 receptor to inhibit NFATc1 expression in OCs while up-regulating Runx2 expression in OBs, mitigating bone mineral loss and microstructural deterioration in PMOP mice.	[148]
	5-HT	Exhibited inhibitory effect on osteoblastic activity and causing bone loss.	[150]
	Melatonin	Mitigated suppression of OB differentiation through activation of the PI3K/AKT and BMP/Smad signaling pathways under GC treatment.	[154]
		Targeted miR-224-5p/SIRT3/AMPK/mTOR axis to alleviate OP progress and activate autophagy in GC-treated hBMSCs.	[155]
		Enhanced BMSC proliferation and osteogenic differentiation and delayed bone loss via the HGF/PTEN/Wnt/β-catenin axis in PMOP.	[157]
		Inhibited the activation of the NLRP3 inflammasome mediated by Wnt/β-catenin signaling pathway to mitigate estrogen deficiency-induced OP.	[158]
		Decreased the level of mitochondrial superoxide via the SIRT1–SOD2 axis in OVX-BMSCs.	[159]
		Facilitated BMSC-driven angiogenesis and osteogenesis–angiogenesis coupling in OVX rats.	[160]
		Accelerated apoptosis of RAW264.7 cells through BMAL1/ROS/MAPK-p38 to improve PMOP.	[161]
		Blocked TRAF6, JNK, PRMT1, and NF-κB signaling to inhibit osteoclastogenesis in OVX mice.	[162]
		Enhanced chromatin accessibility for osteogenic genes to promote osteogenesis of BMSCs and mitigate OP in aging mice.	[59]
		Reduced oxidation levels through the ERK/SMAD and NF-κB pathways to alleviate bone loss in retinoic acid-induced OP model mice.	[163]
		Alleviated H_2_O_2_ induced oxidative damage in MC3T3-E1 cells and enhanced osteogenesis by activating SIRT1.	[164]
		Rescued TNF-α-induced suppression of osteogenesis by modulating interaction between SMURF1 and SMAD1.	[165]
		Relieved hyperglycemia-induced alterations in cellular growth, apoptosis, and calcium influx by inhibiting PERK–eIF2α–ATF4–CHOP signaling axis in OP associated with diabetes.	[166]
RA	QUDA	Restrain proliferation and motility of synoviocytes in a dose-dependent manner.	[183]
	QA	Promoted the proliferation of human fibroblast-like synoviocytes and stimulated mitochondrial respiration and glycolysis.	[181]
	5-HT	Modulated arthritis by balancing the Th17/Treg cell axis and influencing osteoclastogenesis.	[194]
	Melatonin	Heightened production of proinflammatory cytokines in synovial macrophages from RA patients.	[199,200]
		Suppressed Cry1 gene expression to enhance cAMP levels and activate PKA and NF-κB, thereby exacerbating CIA in rat models.	[206,207]
		Mitigated inflammation and modulated thymocyte function related to the G protein–adenyl cyclase–cAMP pathway and Met-Enk release.	[209]
		Reduced TNF-α and IL-1β production in human RA synovial fibroblasts by inhibiting PI3K/AKT, ERK, and NF-κB pathways and enhancing miR-3150a-3p expression.	[89]
		Elevated anti-collagen antibody and IL-1β and IL-6 levels while reducing oxidative stress markers.	[210]
AS	Kyn	Boosted OPG and OCN expressions in AS osteoprogenitors, enhancing bone mineralization and suppressing RANKL-mediated OC differentiation.	[108]
	5-HT	Inverse with pCREB activation in OB-like Saos-2 cells.	[223]
	Melatonin	Positive correlation with OCN and IL-1β in AS.	[224]
		Positive correlation with BASDAI, BASFI, the duration of morning stiffness, and CRP levels.	[225,226]
	IAA	Modulated gut homeostasis and dampening inflammation to manage AS.	[227]
IVDD	Melatonin	Reversed the reduction of trabecular width, increased TGF-β1 expression, and improved disk appearance.	[229]
Scoliosis	Melatonin	Reversed reduced OB proliferation and the development of scoliosis.	[230]
Bone metastases	Kyn	Activated the AhR responsible for SCLC progression and metastasis, and enhanced osteoclastogenesis.	[232]

At the molecular genetic level, Trp molecules are involved in the pathogenesis of diverse disease processes. Glycine-to-Trp substitution in the COL1A1 gene that disrupts proper protein folding and fibril assembly has been documented in osteogenesis imperfecta, a heritable connective tissue disorder [235,236]. A Trp substitution to Arg^103^ was identified through mutation analysis of all 3 collagen IX genes in patients with lumbar disk disease [237]. Besides, a clinically mutation in the vitamin D receptor involving a substitution of Trp by arginine at amino acid 286 has been revealed in vitamin D-resistant rickets, which influenced vitamin D receptor (VDR) trafficking toward the nucleus while selectively abolishing the 24-hydroxylase gene response to 1,25(OH)_2_D_3_ [238]. In summary, the unique biochemical properties of Trp render the vulnerable to pathogenic substitutions in genetic disorders that impair diverse protein functions, from collagen fibrillogenesis to nuclear receptor trafficking.

## Therapeutic Potential of Trp Metabolites in Osteochondral Diseases

Emerging evidence highlights the critical role of Trp metabolism in regulating bone homeostasis, with its metabolites influencing osteoblastogenesis, OC activity, and gut microbiota–bone crosstalk. We further explored the potential of Trp metabolites as therapeutic agents in bone diseases, focusing on their mechanisms of action and translational applications.

Direct administration of key Trp metabolites has shown marked therapeutic potential. IAA and IPA supplementation could not only enhance intestinal barrier function via stimulation of the Wnt/β-catenin signaling pathway but also promote the polarization of M2 macrophages, subsequently secreting substantial amounts of IL-10 to concurrently stimulate osteoblastogenesis and suppress osteoclastogenesis, as evidenced by both in vivo and in vitro studies [170]. RSV, an AhR antagonist, ameliorated the anti-osteoblastogenesis effects of IS through the inhibition of AhR and reversing downstream ERK and p38 MAPK signaling [63]. These findings suggest that simultaneously enhancing beneficial metabolites while inhibiting pathogenic metabolic pathways may serve as promising therapeutic agents for bone disorders by modulating the AhR-mediated gut–bone axis. Besides, a recent study has reported that the inhibition of IDO1 activity not only decreased Treg cell populations and restored cytotoxic T lymphocyte function but also synergistically alleviated tumor microenvironment immune suppression combined with anti-PD-1 antibody treatment, providing a new strategy for the clinical treatment of breast cancer with bone metastasis [239]. The clinical trial published in *JAMA Oncology* evaluating PD-1 blockade with metronomic chemotherapy in sarcomas identified IDO1-mediated immune evasion as a key resistance mechanism. During treatment, predominant infiltration of IDO1-expressing tumor-associated macrophages and significantly elevated plasma Kyn/Trp ratio both indicate robust IDO1 pathway activation that likely counteracts PD-1 inhibition efficacy [240]. IDO1 inhibition demonstrated therapeutic potential across multiple cancer types. A preclinical study revealed that combining the IDO1 inhibitor D1MT with the CXCR4 antagonist AMD3465 significantly delayed breast cancer bone metastasis progression in animal models [241]. Although this dual-targeting strategy showed promise for refractory metastatic breast cancers, its clinical translation requires further validation.

Precision manipulation of gut microbiota offers novel treatment strategies. Specific probiotic strains, including *Lactobacillus*, have demonstrated immunomodulatory effects to achieve osteoprotective function. The richness of *Romboutsia*, *Bacteroides*, and *Alloprevotella genera* effectively alleviated the symptoms of RA in terms of phenotype. Building upon this foundation, specific traditional Chinese medicinal compounds such as *Saussurea* and SIN both demonstrate therapeutic potential for osteochondral disorders by restoring gut microbial equilibrium [214,215]. Alternatively, comparable therapeutic outcomes may be attained through direct probiotic supplementation or fecal microbiota transplantation. Given the effective improvement of metabolic diseases, the microbial community may be a potential therapeutic candidate against osteochondrogenic disorders mediated by the gut–bone axis.

Recent advances in biomaterial engineering have enabled precise spatiotemporal control over Trp metabolite delivery, addressing key limitations of systemic administration. Intra-articular melatonin injections have demonstrated efficacy in mitigating cartilage degeneration in OA animal models. To enhance its therapeutic potential, sustained-release DDS have been developed, such as melatonin-loaded hydrogels and composite scaffolds, which improved cartilage repair by promoting chondrogenic and osteogenic differentiation [242]. Innovative biomaterials, including biphasic hydrogels and injectable gellan gum/lignocellulose nanofibril composites, have been engineered to achieve controlled melatonin release while mimicking the ECM for optimal tissue regeneration [126,136,137]. These material-based strategies synergize with endogenous metabolic pathways, offering solutions to current challenges in metabolite stability, tissue specificity, and treatment duration for bone disorders. Beyond melatonin, the strategic application of biomaterials can be extended to other Trp metabolites, offering potential therapeutic approaches for various bone disorders including bone defects and degenerative conditions.

In the latest research progress, Trp metabolites are probably applied to serve as adjuvant therapies to conventional anti-resorptives or synergize with immune checkpoint inhibitors for treating cancer-related bone diseases. However, several obstacles should be addressed for successful clinical translation. Tissue-specific metabolic variations may cause neurological side effects with systemic administration, and current biomarkers lack precision in evaluating target engagement. Extensive population-based cohort studies are required to elucidate the precise effects of Trp metabolism on bone homeostasis regulation. The development of bone-specific drug delivery platforms, such as bisphosphonate-conjugated nanoparticles or hydroxyapatite-targeting carriers, may optimize local bioavailability of Trp metabolites for treating bone disorders. In the future, advances in metabolomics and gene-editing technologies, as well as the application of artificial intelligence are expected to be tailored to different bone disease subtypes [243], enriching the development of “metabolic microenvironment remodeling” therapies that revolutionized the treatment of chronic bone disorders.

## Conclusion and Perspective

Trp and its related metabolites exhibited intricate roles in the modulation of bone homeostasis, spanning physiological and pathological spectra. Extensive research has established Trp as a precursor to multiple biologically active substances, such as Kyn, serotonin, and indole, among others, emerging as pivotal players in the modulation of OB, OC and chondrocyte function. The metabolites influenced osteochondral cell activities encompassing proliferation and differentiation through diverse pathways, and their activation implicated in the etiology and pathology of osteochondral destruction diseases, including OA, OP, RA, and AS, has been progressively unveiled. Additionally, the gut microbiota contributed to intestinal homeostasis by metabolizing Trp into molecules like indole and its derivatives, which modulated the equilibrium between pro-inflammatory and anti-inflammatory cytokines. Trp metabolites interact with certain receptors such as AhR, potentially mitigating bone loss through a gut–bone axis, underscoring the multifaceted influence of Trp derived from gut microbiota on skeletal health. Furthermore, variations in Trp levels were discernible across a spectrum of bone-related disorders, demonstrating its capacity to function as a modulatory factor or a biomarker in disease processes. Investigations into materials based on Trp metabolites have also presented promising therapeutic outcomes in animal models.

Although the individual roles of Trp metabolites in bone biology have been increasingly studied, the interactions between these metabolites and their combined effects on bone cells remain poorly understood. The complexity of metabolic networks demonstrates that these metabolites may act synergistically, antagonistically, or in a context-dependent manner to regulate bone homeostasis. For instance, the Kyn pathway and indole derivatives may compete for the same precursor, creating a metabolic balance that could influence OB and OC activity. Elevated Kyn levels, often associated with inflammatory conditions, might suppress osteogenesis, while indole derivatives such as IPA could counteract this effect by promoting mitochondrial function and OB differentiation. Moreover, AhR, a common target for both Kyn and indole metabolites, presents a potential point of interaction. While Kyn activation of AhR has been linked to pro-inflammatory and osteoclastogenic effects, certain indole derivatives may exert anti-inflammatory and osteoprotective effects through the same receptor. This duality highlights the need to investigate how the relative abundance and timing of these metabolites shape AhR signaling and downstream bone cell behavior. Additionally, serotonin may interact with Kyn and indole pathways in bone regulation. For example, serotonin’s peripheral effects on bone formation could be modulated by the availability of Trp for its synthesis, which is influenced by the activity of the Kyn and indole pathways. These intricate interactions suggest that the metabolic network is not only a collection of independent pathways but also a dynamic system where metabolites influence each other’ effects on bone cells. Despite the theoretical framework, direct experimental evidence on these interactions is currently lacking. Future studies should focus on coculture systems, multi-omics approaches, and in vivo models to dissect the interplay between Trp metabolites and their combined effects on bone biology. Understanding these interactions could provide novel therapeutic strategies for osteochondral disorders by targeting the metabolic network as a whole rather than individual pathways.

Moreover, current literature indicated the dual effect of Trp metabolites in maintaining bone homeostasis, with their effects potentially shaped by a wide array of factors such as dosage and the timing of administration, the choice of experimental animal models, and inherent interindividual variability. The precise mechanism of modulation in physiological activity and pathological progression may involve multiple signaling pathways. A comprehensive framework to conclusively explain the diverse impacts of Trp metabolites on bone health is still in development. In addition, the recognition of the interplay between Trp metabolism and intestinal flora in bone homeostasis is a promising frontier in biomedical research. The gut microbiota exerts a systematic and remote regulatory influence over bone metabolism, with associations with multiple elements such as integrity of the intestinal mucosal barrier and the dynamic equilibrium of the immune system. Despite this extraordinary potential, the field suffers from critical knowledge gaps, where clinical studies that directly address indole–bone interactions remain virtually nonexistent. The striking disconnection between demonstrated potency such as nanomolar-range efficacy in vitro and limited investigation presents both a challenge and opportunity. The precise molecular mechanisms underlying the gut–bone axis and the potential therapeutic targets remain to be further investigated. Urgent priorities include establishing dose–response relationships, identifying optimal producer strains, and developing targeted delivery systems to harness these compounds’ exceptional bioactivity.

Subsequent research may concentrate on clarifying the intricate regulatory network between Trp metabolism, gut microbiota, immune function, and endocrine balance in osteochondral destruction diseases. The continued investigation of its role, coupled with the exploration of microbiome-targeted therapies, holds the promise of advancing our ability to treat and prevent bone pathologies. The advancement of innovative diagnostic techniques and treatment strategies that target Trp and its derivative-mediated signaling pathway could revolutionize the management of bone disorders. The clinical application of Trp metabolites as biomarkers in osteochondral destruction diseases still demands validation through large-scale clinical studies ensuring their reliability and effectiveness.

## Data Availability

The data that support the findings of this study are available from the corresponding author upon reasonable request.
